# Genetic Interaction Mapping Reveals a Role for the SWI/SNF Nucleosome Remodeler in Spliceosome Activation in Fission Yeast

**DOI:** 10.1371/journal.pgen.1005074

**Published:** 2015-03-31

**Authors:** Kristin L. Patrick, Colm J. Ryan, Jiewei Xu, Jesse J. Lipp, Kelly E. Nissen, Assen Roguev, Michael Shales, Nevan J. Krogan, Christine Guthrie

**Affiliations:** 1 Department of Biochemistry and Biophysics, University of California, San Francisco, California, United States of America; 2 Systems Biology Ireland, University College Dublin, Dublin, Ireland; 3 Department of Cellular and Molecular Pharmacology, University of California, San Francisco, California, United States of America; 4 California Institute for Quantitative Biosciences, QB3, San Francisco, California, United States of America; 5 J. David Gladstone Institutes, San Francisco, California, United States of America; University of Colorado Denver, UNITED STATES

## Abstract

Although numerous regulatory connections between pre-mRNA splicing and chromatin have been demonstrated, the precise mechanisms by which chromatin factors influence spliceosome assembly and/or catalysis remain unclear. To probe the genetic network of pre-mRNA splicing in the fission yeast *Schizosaccharomyces pombe*, we constructed an epistatic mini-array profile (E-MAP) and discovered many new connections between chromatin and splicing. Notably, the nucleosome remodeler SWI/SNF had strong genetic interactions with components of the U2 snRNP SF3 complex. Overexpression of SF3 components in *ΔSWI/SNF* cells led to inefficient splicing of many fission yeast introns, predominantly those with non-consensus splice sites. Deletion of SWI/SNF decreased recruitment of the splicing ATPase Prp2, suggesting that SWI/SNF promotes co-transcriptional spliceosome assembly prior to first step catalysis. Importantly, defects in SWI/SNF as well as SF3 overexpression each altered nucleosome occupancy along intron-containing genes, illustrating that the chromatin landscape both affects—and is affected by—co-transcriptional splicing.

## Author Summary

It has recently become apparent that most introns are removed from pre-mRNA while the transcript is still engaged with RNA polymerase II (RNAPII). To gain insight into possible roles for chromatin in co-transcriptional splicing, we generated a genome-wide genetic interaction map in fission yeast and uncovered numerous connections between splicing and chromatin. The SWI/SNF remodeling complex is typically thought to activate gene expression by relieving barriers to polymerase elongation imposed by nucleosomes. Here we show that this remodeler is important for an early step in splicing in which Prp2, an RNA-dependent ATPase, is recruited to the assembling spliceosome to promote catalytic activation. Interestingly, introns with sub-optimal splice sites are particularly dependent on SWI/SNF, suggesting the impact of nucleosome dynamics on the kinetics of spliceosome assembly and catalysis. By monitoring nucleosome occupancy, we show significant alterations in nucleosome density in particular splicing and chromatin mutants, which generally paralleled the levels of RNAPII. Taken together, our findings challenge the notion that nucleosomes simply act as barriers to elongation; rather, we suggest that polymerase pausing at nucleosomes can activate gene expression by allowing more time for co-transcriptional splicing.

## Introduction

Recent work has uncovered extensive crosstalk amongst chromatin, transcription and RNA processing machineries. Changes to chromatin typically involve nucleosomes—histone octomers wrapped by approximately 147 nucleotides of DNA. We now know that nucleosomes are enriched in exons relative to introns [1,2] and that intronic and exonic histones are marked differentially [3] suggesting that nucleosomes may be involved in defining intron/exon junctions and that certain histone marks might influence splicing decisions. Importantly, nucleosomal contacts with DNA are constantly modulated by ATP-dependent chromatin remodeling complexes (e.g. SWI/SNF, Ino80, and RSC), that function to deposit, remove, and/or slide nucleosomes [4]. Although primarily studied in the context of regulation of transcription, nucleosome remodeling is also likely to influence splicing in numerous ways: altering RNA polymerase II elongation rates, promoting RNAPII pauses, and/or recruiting the spliceosome to chromatin via protein-protein interactions (Reviewed in [5]).

Most of what we know about co-transcriptional splicing regulation comes from studies of alternative splicing in mammals, in which histone modifications (e.g. H3K36me3 [6]) and chromatin remodelers (e.g. SWI/SNF [7,8]) have been shown to modulate exon skipping (reviewed in [9,10]). However, most of this work has focused on a small set of alternatively spliced reporter genes and has not revealed mechanistic insights into how specific steps of spliceosome activation and/or catalysis can be influenced by changes to chromatin. Additionally, while there is good evidence that splicing can direct histone H3K36 tri-methylation [11,12] and H3K4 tri-methylation [13], we still know very little about how splicing may more broadly influence chromatin states.

Despite the relatively simple intron/exon architecture of the *Saccharomyces cerevisiae* genome, there is mounting evidence that chromatin and transcription also play an important role in promoting splicing in budding yeast. Specifically, ubiquitination of histone H2B has been linked to spliceosome assembly and function [14,15] and histone acetylation has been shown to promote pre-catalytic spliceosome assembly [16,17]. RNA polymerase speed has also been correlated with splicing efficiency in *S*. *cerevisiae* [18,19]. Taken together, these results suggest that many of the fundamental mechanisms linking chromatin and splicing are conserved throughout evolution.

Here, we present work showing extensive connections between pre-mRNA splicing and chromatin in the fission yeast, *Schizosaccharomyces pombe*. This yeast is a uniquely attractive model organism—it is as genetically tractable as its distant cousin, *S*. *cerevisiae*, but much more metazoan-like in its dependence on splicing; over half of fission yeast genes contain an intron and of those, half contain more than one intron [20]. Though examples of alternative splicing are rare to date [21,22] it is notable that many fission yeast introns have non-consensus splice sites [23], suggesting that so-called constitutive introns vary in efficacy of removal. Using high-throughput genetic interaction mapping, we uncovered multiple connections between the chromatin and splicing machineries. Importantly, we found that deletion of SWI/SNF nucleosome remodeling complex components left cells particularly sensitive to overexpression of the SF3 subcomplex of the U2 snRNP. We present evidence that SWI/SNF contributes to splicing catalysis by promoting recruitment of the Prp2 ATPase, which acts to destabilize SF3 immediately prior to first step catalysis. Our data provide a functional role for SWI/SNF in the splicing of introns with weak splice sites and promote the idea that nucleosomes, while serving as barriers to RNAPII elongation, may actually promote co-transcriptional splicing.

## Results

### Generation of a pre-mRNA splicing E-MAP in *S*. *pombe*

In order to define the genetic interaction space of all pre-mRNA splicing factors in fission yeast, we created a set of mutants in which each gene involved in splicing was individually removed or perturbed. We first manually compiled a list of 81 genes whose products have either been experimentally implicated in fission yeast splicing or whose orthologs have been functionally characterized as splicing factors in other species (S2 Table). As was originally described in Roguev et al., (2008), for genes annotated as non-essential, we systematically replaced the open reading frame with a NAT resistance cassette to create deletion strains. For essential genes (which encode the majority of splicing factors in *S*. *pombe*), we created DAmP alleles [24] by replacing the 3’UTR with a NAT resistance cassette. DAmP alleles have been successfully used in the past to perturb mRNA levels of essential genes in order to shed light on their function in high-throughput genetic and drug screens [25]. Using the Pombe Epistasis Mapper (PEM) system our group developed [26], we screened 74 *S*. *pombe* splicing factors (several splicing mutants were too sick to be propagated through the E-MAP screen; see S2 Table) against a fission yeast mutant library containing more than 2,000 non-essential deletions (library as described in [27]). This collection represented virtually every major known biological process in the cell, creating a Splicing E-MAP with approximately 120,000 pairwise measurements. Positive genetic interactions between two mutants (> +2.0) represent epistasis or suppression, while negative genetic interactions (< -2.5) represent synthetic sickness, or in some cases, synthetic lethality. We present this data in S1 Table.

**Fig 1:**
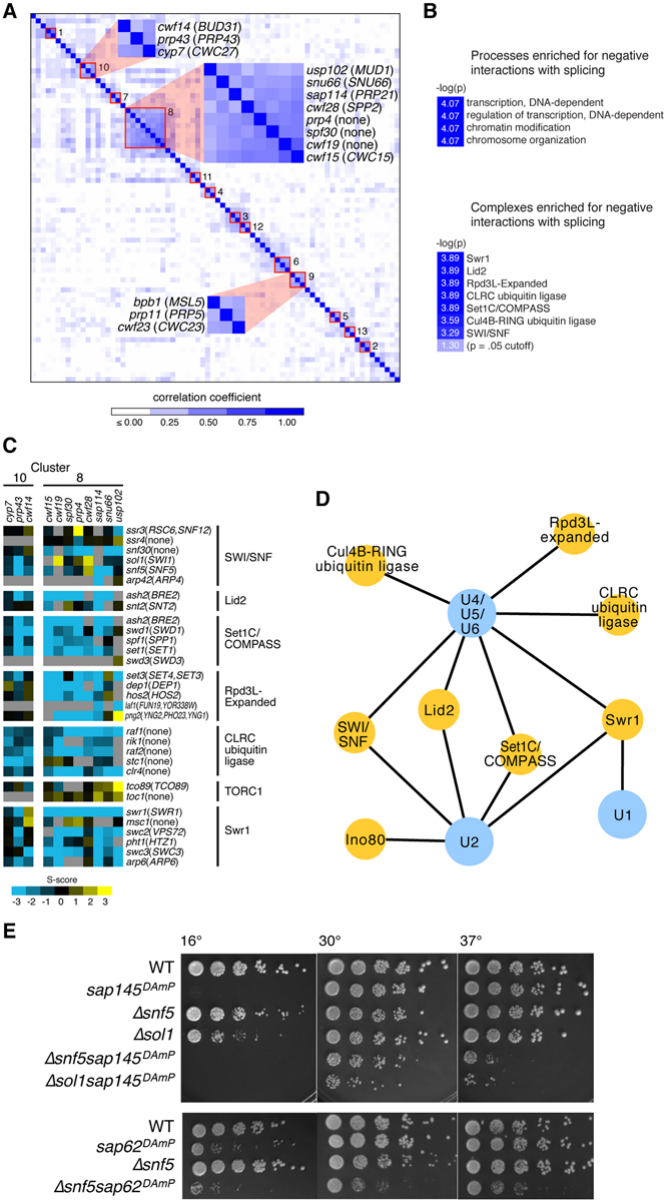
Splicing has strong negative genetic interactions with the chromatin machinery. (A.) Hierarchical clustering of genetic interaction profile correlations between each pair of splicing factors in the Splicing E-MAP. Negative correlations were scored as zero in the heatmap. Red boxes indicate clusters of splicing factors whose correlation coefficients > 0.3. Clusters 8, 9, and 10 are highlighted. (B.) Processes (top) and complexes (bottom) enriched for negative interactions with splicing in the Splicing E-MAP. Light blue box indicates color of significance cut-off. Processes were defined using the *S*. *pombe* GO Slim database and complexes were taken from the *S*. *pombe* cellular component Gene Ontology list (PomBase). Bonferonni corrected p value <0.0001 for processes and ≤0.0005 for complexes. (C.) Genetic interactions of Splicing Cluster 8 and 10 with the chromatin machinery. Blue indicates negative genetic interactions; yellow indicates positive; grey indicates no data. (D.) Splicing U snRNPs (blue) enriched for negative genetic interactions with chromatin complexes (yellow). U snRNPs were defined as listed in S2 Table. Bonferonni corrected p-value <0.05, False Discovery Rate <10%. (E.) Serial dilutions (1:5) of WT, single, and double mutants grown at 16°, 30° and 37°C.

Previous work (summarized in [28]) has shown that genes belonging to the same complex or pathway tend to have similar genetic interaction profiles. To gain an unbiased overview of the splicing factors in our E-MAP we clustered them according to the similarity of their genetic interaction profiles, i.e. based on how individual splicing factor mutants genetically interact with all factors represented in the deletion library. Several clusters (correlation coefficient > 0.3, see methods) emerged containing splicing factors from multiple steps of the splicing cycle (Fig. 1A and S2 Table). Consistent with our understanding of early spliceosome assembly, our E-MAP revealed a high degree of correlation between a pair of splicing factors, prp11 (PRP5; where appropriate, *S*. *cerevisiae* orthologs will be listed parenthetically) and the branchpoint binding protein bpb1 (MSL5/BBP1), which are both known to function at the step of intron recognition (Fig. 1A, Cluster 9). Interestingly, cwf23 (CWC23) is also part of Cluster 9, perhaps indicating a role for this DNAJ domain-containing protein at an early step of spliceosome assembly.

Generally, splicing genetic interaction profile clusters did not breakdown into U snRNPs, the small nuclear ribonuclear protein complexes that assemble stepwise to form a functional spliceosome. Interpretation of the E-MAP genetic interaction profile correlation scores for splicing factors is complicated by the fact that most of them are essential and thus are represented in the E-MAP as DAmP alleles. Integration of the DAmP cassette is not expected to affect expression of all genes equally. Indeed, as was the case in the *S*. *cerevisiae* RNA processing E-MAP [29], many of the *S*. *pombe* DAmP strains showed little growth defect and had weak genetic interaction profiles, making clustering uninformative in some cases and resulting in clusters comprised mostly of mutants with strong genetic interaction profiles. Despite these caveats, our observation that certain “early” splicing factors and “late” splicing factors had similar genetic interaction profiles reinforces the dynamic nature and functional complexity of the spliceosome.

### Genetics reveal myriad connections between splicing and chromatin machineries

We next asked what cellular pathways were enriched for positive or negative genetic interactions with splicing as a whole. Positive genetic interactions can identify genes whose products act in the same biological pathway, or in some cases, form a protein complex. Negative genetic interactions are often observed between two genes whose products act in parallel pathways that lead to a common biological outcome [28]. Strikingly, of all the pathways represented in the E-MAP [27], splicing was specifically enriched for negative genetic interactions with factors related to “transcription,” “regulation of transcription,” “chromatin modifications” and “chromosome organization” (Fig. 1B, methods). Looking next at negative genetic interactions between splicing and known protein complexes, we saw additional evidence for significant crosstalk between splicing and chromatin: *S*. *pombe* splicing mutants had strong negative genetic interactions with chromatin remodeling factors, such as the histone exchange complex SWR1, and histone modifying factors, including the histone demethylase Lid2 and the histone deacetylase complex Rpd3L (Fig. 1B). Interestingly, there appears to be some specificity regarding which splicing factors have genetic interactions with particular chromatin complexes. For example, splicing factors in Cluster 8 exhibited strong negative genetic interactions with Rpd3L factors, supporting a role for histone deacetylation in promoting splicing, while Cluster 10 factors did not (Fig. 1C).

Although the composition of U snRNPs was not immediately evident from the clustering, we still wanted to know whether there was any specificity in how different U snRNPs interacted genetically with chromatin complexes. We found that several chromatin complexes were enriched for negative genetic interactions with multiple U snRNPs (e.g. Set1/COMPASS, Lid2, SWI/SNF, and SWR1) (Fig. 1D). However, a smaller subset of chromatin complexes showed enrichment for negative genetic interactions with a limited set of U snRNPs—for example, tri-snRNP (U4/U5/U6) showed strong negative interactions with two ubiquitin ligase complexes (CLRC and Cul4B-RING) and the U1 snRNP was strongly negative with the SWR1 histone exchange complex. Notably, the nucleosome remodelers SWI/SNF and Ino80 were particularly enriched for negative interactions with the U2 snRNP (Fig. 1D), as well as with downstream tri-snRNP components. SWI/SNF and Ino80 are ATP-dependent chromatin remodeling complexes whose activities include ordering and reorganizing nucleosomes on the DNA template in order to regulate RNA polymerase II elongation. A subunit of SWI/SNF has been previously shown to influence alternative splicing in mammals [7], although its mechanism for doing so remains unclear. We therefore set out to elucidate the precise interplay between SWI/SNF and splicing in fission yeast.

We identified two splicing factors with strong negative genetic interactions with components of SWI/SNF: *sap145* and *sap62*. Sap145 (CUS1) is a component of SF3b, a subcomplex of U2 that associates with the pre-mRNA branchpoint region [30]. *Sap62* (PRP11) is a component of the SF3a complex, a group of three conserved proteins that binds to SF3b and also makes contacts with the intron branchpoint. Because both *sap145* and *sap62* are essential genes in *S*. *pombe*, they were represented in the E-MAP as DAmP alleles. In order to confirm the E-MAP results, as well as to assess temperature sensitivities, we crossed these DAmP alleles with two non-essential SWI/SNF deletions, *Δsnf5* and *Δsol1*. *Snf5* is a core component of SWI/SNF while *Sol1* (SWI1) is an ARID (**A**T-**r**ich **i**nteraction **d**omain)-containing protein that makes specific contacts with DNA [31]. As predicted by the negative genetic interaction score in the EMAP, combining the SF3 DAmP alleles with Δ*snf5* or Δ*sol1* caused synthetic sickness at all temperatures, most severely at 37°C and 16°C (Fig. 1E). Results from the E-MAP also showed negative genetic interactions between SWI/SNF and other components of SF3, namely *sap114* and *prp10* (PRP21 and HSH155, respectively) (S1 Fig), further suggesting there might be specific interplay between the SWI/SNF nucleosome remodeling complex and the U2 snRNP. We thus set out to understand the molecular phenotype behind the synthetic sickness in our SWI/SNF-SF3 double mutants.

### SWI/SNF-SF3 double mutants have a broad yet intron-specific splicing defect

**Fig 2:**
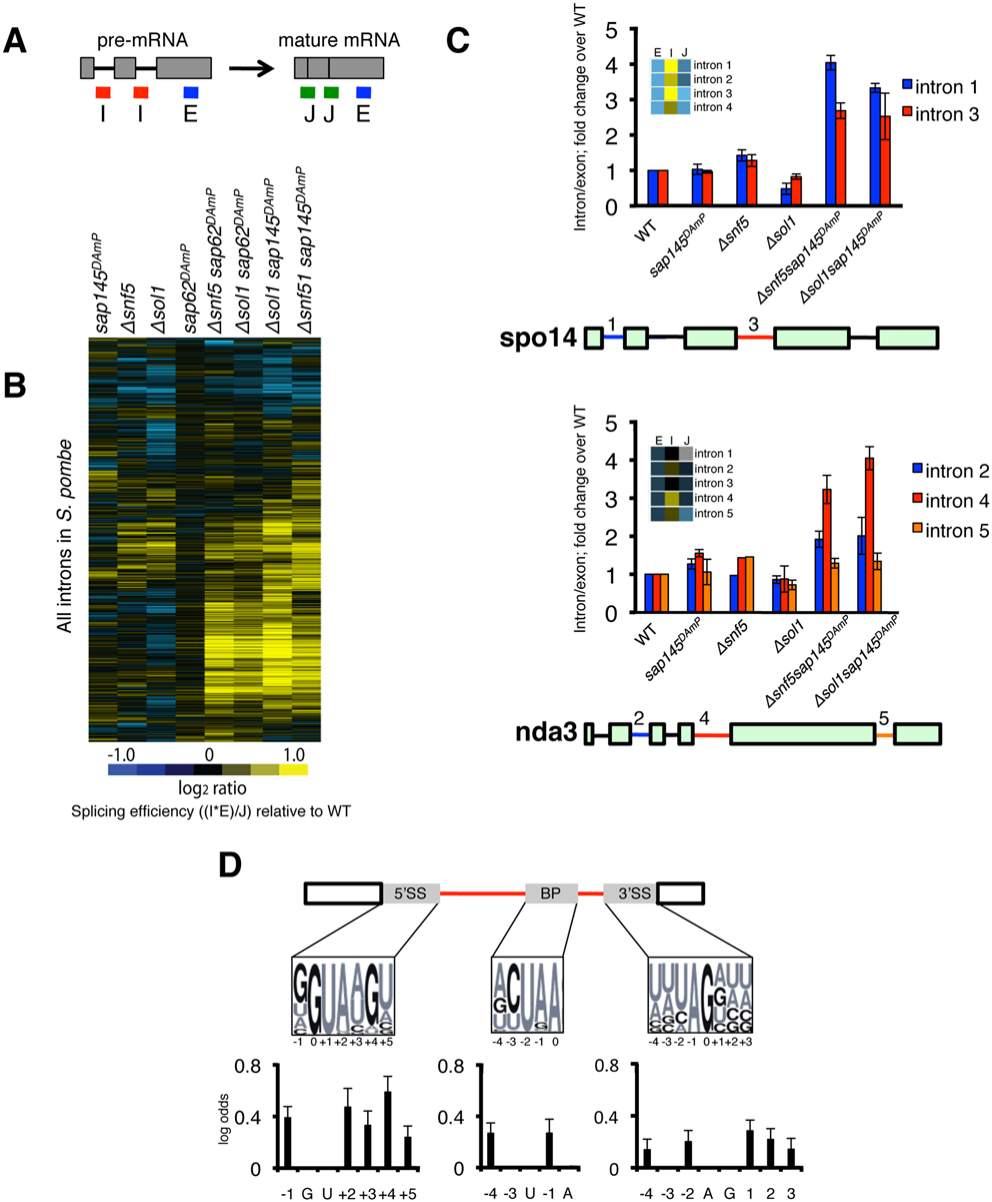
SWI/SNF-SF3 double mutants have a splicing defect. (A.) Diagram representing probes on microarrays. (B.) Splicing microarray of single (*Δsnf5*, *Δsol1*, *sap145* ^*DAmP*^, *sap62* ^*DAmP*^) and double mutants (*Δsnf5 sap62* ^*DAmP*^, *Δsol1 sap62* ^*DAmP*^, *Δsnf5 sap145* ^*DAmP*^, *Δsol1 sap145* ^*DAmP*^). Heatmap represents the log_2_FC for most introns in *S*. *pombe*. Log_2_FC is the ratio of each of the mutant features relative to wild-type, expressed as a splicing efficiency value ((intron*exon)/junction), transformed to log_2_ space. (C.) RT-qPCR validation of microarray. Bars represent fold change of intron/exon amplicons normalized to WT. Error bars represent ±SEM of the average of 3–4 biological replicates. Heatmap scale is same as in Fig. 2B. (D.) Changes to consensus splice sites increase likelihood of intron retention in *Δsol1sap145* ^*DAmP*^ double mutant. Logos represent consensus site sequences for the 5’SS, BP and 3’SS. Probability of a specific nucleotide change increasing the likelihood of retention in the double mutant is represented as log odds (positive log odds at a given position indicates that non-consensus nucleotides correlate with intron retention). Affected introns were defined as having a corrected p value of < 0.05. Dark nucleotides in the consensus site logos are GCs, lighter letters are AUs.

In order to ascertain whether defective splicing was responsible for the growth defect in the SWI/SNF-SF3 double mutant strains, we performed genome-wide splicing microarray analysis. These arrays contain oligonucleotide probes specific for the exon, intron, and exon-exon junction for nearly every canonically spliced intron in fission yeast—allowing us to compare the levels of total, pre-mRNA and mature mRNA, respectively, for any given mutant strain against an isogenic wild-type (Fig. 2A). The experiment was performed as a competitive two-color hybridization and results are reported as the log_2_ fold change (logFC) in mutant RNA compared to wild-type RNA (Lipp et al., in preparation), with yellow in the Fig. 2B heatmap signifying accumulation of pre-mRNA relative to mature mRNA. We observe little accumulation of pre-mRNA in any of the single mutants (*sap145* ^*DAmP*^, *sap62* ^*DAmP*^, *Δsnf5*, *Δsol1*, Fig. 2B), which we interpret to mean there is little to no change in splicing efficiency in these strains. However, upon combining mutations in splicing factors and SWI/SNF, we see a broad yet intron-specific accumulation of pre-mRNA (Fig. 2B). Average hierarchical linkage clustering reveals a high degree of correlation between sets of double mutants: SWI/SNF-SF3a double mutants have a correlation of 0.85 and the SWI/SNF-SF3b double mutants have a correlation of 0.76 (S2 Fig). Together, the SWI/SNF-SF3 strains correlate to a relatively high degree (0.65), indicating that similar introns are affected in all SWI/SNF-SF3 double mutant backgrounds. To quantify the exacerbated splicing defect in the double mutants, we imposed a significance cut-off of a logFC of 0.5 and calculated the number of introns that were spliced poorly (in yellow) or spliced better (in blue) for each of the single and double mutant strains (S2 Fig). Again, we observed few changes in the number of introns retained in the single mutants (<300 in each case), but many more retained introns in the SWI/SNF-SF3 double mutants (>1000 in most cases), and of those, the double mutants had more introns retained to higher degrees (logFC > 1.5, in darker yellow). While these results are consistent with a splicing defect, we cannot rule out that combination of SWI/SNF deletion with the SF3 DAmP alleles induces changes in rates of pre-mRNA decay by nuclear and/or cytoplasmic surveillance pathways.

We validated our microarray results by reverse transcriptase quantitative PCR (RT-qPCR), choosing two representative multi-intron containing genes, spo14 and nda3, using primers that amplify the intron-exon junction (intron) and the exon (total) (Fig. 2C). Again, we only saw intron accumulation in the double mutant strains, relative to wild type, providing further evidence that the splicing defect is only seen when both SF3 and SWI/SNF are mutated. Interestingly, when we looked at a multi-intron containing gene like nda3, we saw that intron 4 accumulated to much higher levels in the double mutants than did other introns in the same gene, indicating that the splicing defect is intron-specific, even when affected introns are very close to other introns in the gene.

The specificity of intron retention suggested to us that there might be cis-splicing signals that make an intron more or less sensitive to the SWI/SNF-SF3 mutations. To interrogate this, we computationally assessed the contribution of different intron features to the splicing defect. While the cis-splicing signals of *S*. *pombe* introns are less constrained then those of *S*. *cerevisiae*, there are consensus sequences maintained by the majority of *S*. *pombe* introns. Analysis of the microarray data revealed that deviation from consensus in those sequences (the 5’ splice site (5’SS), the branchpoint (BP), and the 3’ splice site (3’SS)) increased the likelihood that an intron would be retained in the *Δsol1sap145* ^*DAmP*^ double mutant strain (Fig. 2D). Interestingly, we observed that splicing in the double mutant is most sensitive to changes to the 5’SS, which may reflect cooperativity between SWI/SNF and the U6 snRNA’s ability to engage the 5’SS, perhaps shedding light on the negative genetic interactions between SWI/SNF and the tri-snRNP from the E-MAP (Fig. 1D). Looking back to the introns that accumulated in our RT-qPCR experiments, we saw that intron 3 of spo14 has a rare 5’SS (GTATGC, used by less than 200 introns in *S*. *pombe*) and intron 4 of nda3 contains a disfavored AAG 3’SS (YAG is preferred, where Y = pyrimidine). No intron features significantly increased or decreased probability of retention in any of the single mutant strains, which is consistent with the overall lack of splicing phenotype in those strains. Several of the mutants had a population of introns whose splicing was improved (in blue on S2 Fig), but there were insufficient numbers for statistical analysis of enriched cis-splicing signals. Taken together, this analysis suggests that non-consensus introns have a unique requirement for interplay between the SWI/SNF nucleosome remodeler and SF3, and surprisingly, suggests these two machineries may play a concerted role in splicing.

### Overexpression of SF3 is synthetic sick with SWI/SNF

**Fig 3:**
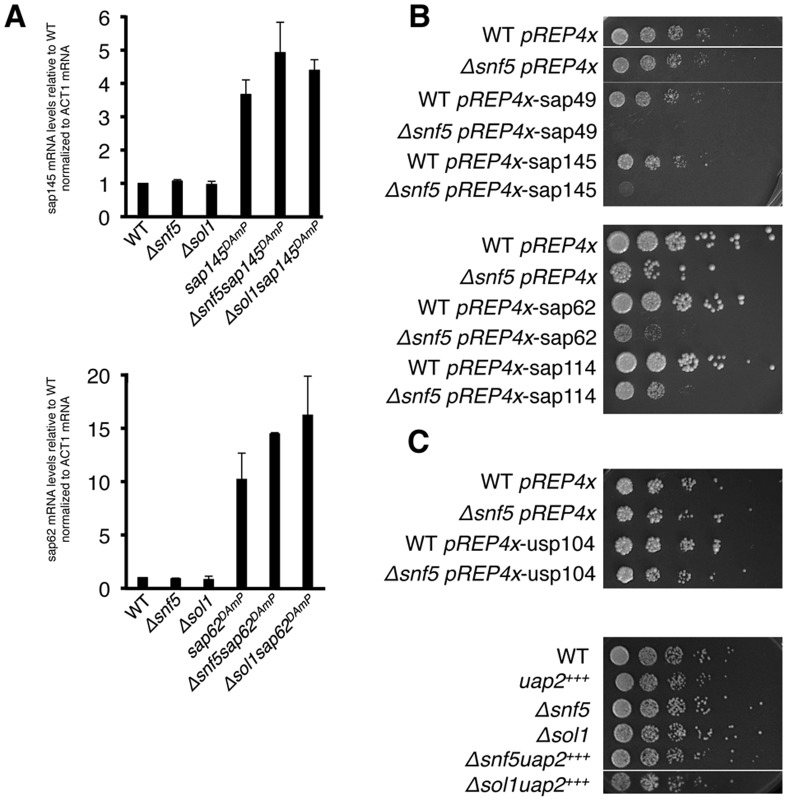
Overexpression of sap145 and sap62 is synthetic sick with SWI/SNF. (A.) RT-qPCR of *sap145* and *sap62* mRNA levels in single and double mutants strains. Bars represent fold change of *sap145* or sap62 signal over WT, normalized to *act1* mRNA. (B.) Serial dilutions (1:5) of WT and *Δsnf5* ectopically overexpressing SF3b (top) and SF3a (bottom) factors grown at 30°C. (C.) Serial dilutions (1:5) of WT and *Δsnf5* ectopically overexpressing usp104 (PRP40) (top) and DAmP overexpression of uap2(CUS2) crossed with *Δsnf5 and Δsol1* (bottom).

In order to look more closely at the mechanistic causes of the splicing defect in the SWI/SNF-SF3 double mutant strains, we needed to understand the effect of the DAmP cassette on SF3 transcript levels. Generally, the DAmP cassette is thought to compromise mRNA processing and stability by removing a gene’s 3’UTR, thus creating hypomorphic alleles. To interrogate the expression level of the *sap145* and *sap62* DAmP alleles, we performed RT-qPCR on total RNA isolated from each mutant strain. Unexpectedly, we found significantly higher levels of mRNA for both *sap145* and *sap62*, indicating that these are in fact, overexpression alleles (Fig. 3A). While this result contradicts the paradigm of 3’UTR disruption creating hypomorphic alleles (DAmP = decreased abundance by mRNA perturbation), we propose that DAmP allele overexpression may be a general phenomenon for genes encoding RNA binding proteins, many of which are known to negatively regulate their own expression through binding to their 3’UTRs [32]. Removal of snf5 and sol1 alone did not affect *sap145* or *sap62* mRNA levels, although the overexpression appeared slightly exacerbated in the double mutant background. Importantly, overexpression of the *sap145* ortholog CUS1 in *S*. *cerevisiae* has previously been purported to promote a tighter association between SF3b, SF3a, and Prp5 [33].

As measuring protein levels would require tagging the proteins, most simply at either the N- or C-terminus, and would likely unintentionally re-create the DAmP alleles, we set out to ectopically overexpress *sap145* and *sap62* and ask whether this recapitulated the synthetic sick phenotype seen with the DAmPs when combined with *Δsnf5*. *Sap145* and *sap62*, along with *sap49* and *sap114*, other components of SF3b and SF3a, respectively, were cloned into the *pREP4x* expression vector, which is under the control of the thiamine-repressible *nmt1* promoter [34] Overexpression of any of the SF3 factors in WT cells did not confer any growth defect while overexpression in the *Δsnf5* strain background resulted in synthetic sickness (Fig. 3B). We took this as confirmation that the DAmP cassette caused overexpression of *sap145* and *sap62* and that *overexpression* of these splicing factors causes synthetic sickness and intron retention when combined with SWI/SNF mutants. S3B Fig shows levels of SF3 factor overexpression in the pREP4x strains, which are comparable to levels induced by the DAmP cassette. As controls, we also overexpressed a non-SF3 U2 factor, *uap2* (CUS2), and a U1 snRNP factor, *usp104* (PRP40), in WT and *Δsnf5* cells. We did not observe any specific genetic interaction between *Δsnf5* and overexpression of uap2 or *usp104*, indicating that merely overexpressing any early splicing factor is not sufficient to induce a growth defect in *Δsnf5* cells (Fig. 3C). We also overexpressed *sap145* in other chromatin mutants (*Δswr1(SWR1)*, Δ*pht1(HTZ1)* and *Δset1(SET1)*) and observed no synthetic sickness (S3 Fig), consistent with a specific interaction between deletion of SWI/SNF and SF3 overexpression. Because we only see intron accumulation when the two mutations are combined, we reasoned that SWI/SNF might contribute to an SF3-dependent step of splicing, possibly by promoting spliceosome recruitment and/or pre-catalytic spliceosome rearrangements. Because we observed no synthetic sickness between *Δsnf5* and mutants of the Prp5 RNA-dependent ATPase (*Prp11* in *S*. *pombe*) (S3 Fig) [35], we hypothesized that SWI/SNF may act at a step of splicing that follows spliceosome assembly.

### SWI/SNF influences splicing at the Prp2 step

**Fig 4:**
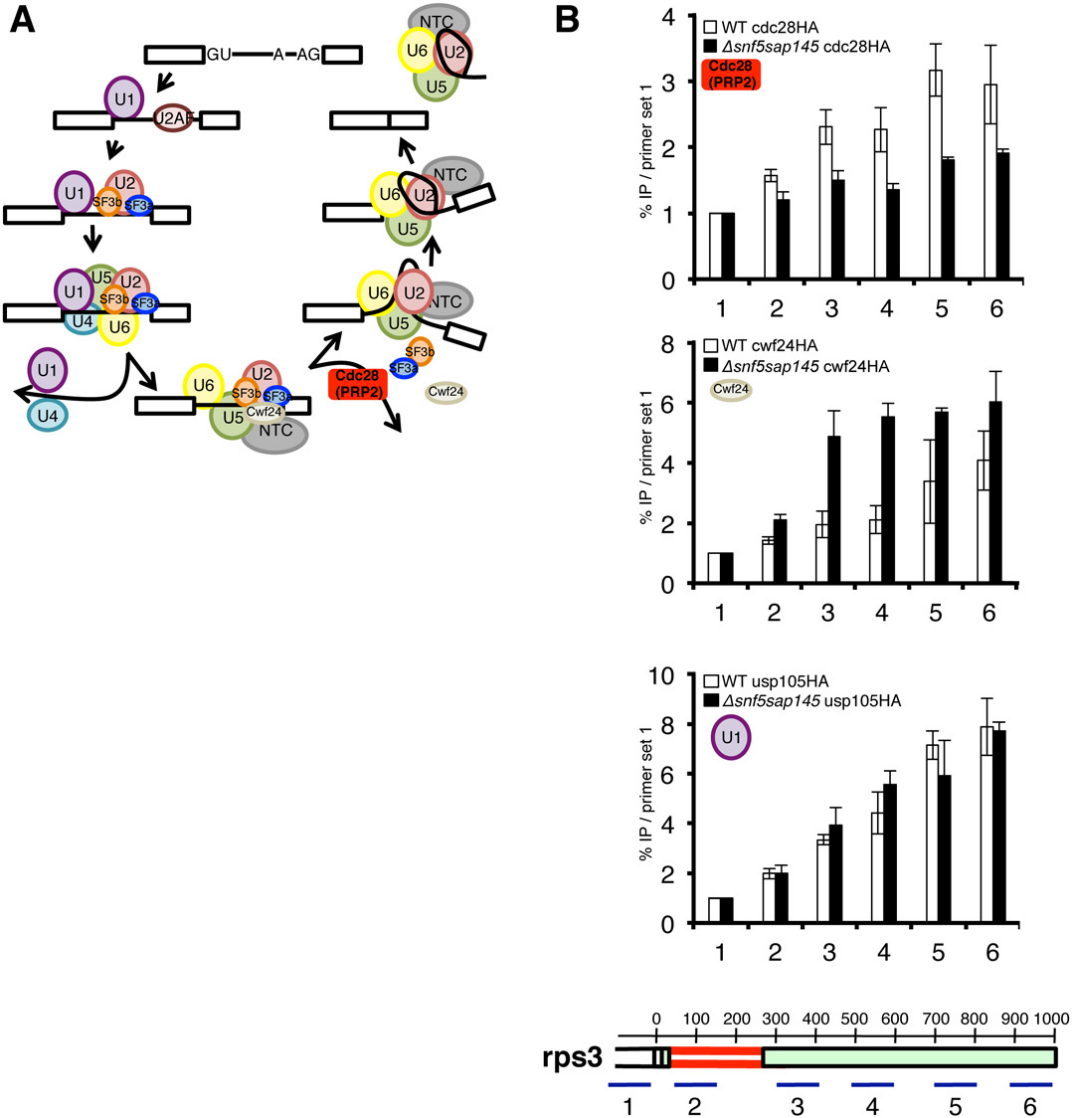
Deletion of SWI/SNF results in decreased recruitment of Cdc28 (PRP2) and retention of Cwf24 (CWC24). (A.) Schematic diagram of the splicing cycle, highlighting the Prp2 ATPase (Cdc28) (red). (B.) ChIPs with α-HA antibody in WT and *Δsnf5sap145* ^*DAmP*^ expressing HA-tagged Cdc28 (PRP2), Cwf24 (CWC24) and Usp105 (PRP39). Bars represent %IP (IP/input) normalized to the % IP at primer set 1 (upstream of promoter/background amplicon). Error bars are ±SEM for each primer set, n = 2 or 3 biological replicates. Gene diagram below indicates approximate location of qPCR amplicons along *rps3* with basepairs annotated.

The microarray results and synthetic sick phenotype suggested that SWI/SNF and SF3 contribute to splicing in a concerted fashion, perhaps both influencing a particular step in the splicing cycle. Having confirmed that our SF3 DAmP alleles were being overexpressed, we wanted to look at specific steps of splicing that might be affected by an overabundance of SF3 components. Importantly, SF3 needs to be destabilized by the ATPase Prp2 prior to the first step of splicing in order for the branchpoint adenosine on the pre-mRNA intron to be available for nucleophilic attack (Fig. 4A) [36–38]. We therefore hypothesized that having an excess of SF3 factors could disfavor first step catalysis and would exacerbate a splicing defect occurring at the Prp2 step.

To follow spliceosome rearrangements in the context of chromatin, we performed splicing factor chromatin immunoprecipitation (ChIP) (Fig. 4B), which has been used successfully in the past as a read-out for the relative timing and efficiency of splicing factor recruitment [39,40]. We chose several intron-containing genes to ChIP based on the following criteria: 1.) The gene had one or more introns whose splicing was defective in a SWI/SNF-SF3 double mutant by microarray, RT-qPCR or both, 2.) The gene was highly expressed, 3.) The gene had introns long enough (>200nt) to provide enough spatial resolution for us to detect co-transcriptional loading and rearrangements of the individual spliceosome factors.

To test the hypothesis that the Prp2 step is affected in the double mutant, we HA-tagged cdc28 (PRP2) and performed ChIP qPCR on at set of representative genes. Strikingly, we found levels of Cdc28 to be significantly lower in the *Δsnf5/sap145* ^*DAm*P^ strain compared to wild type at three different genes with distinct intron architectures: *rps3*, *dbp2* and *SPBC660*.*16* (Fig. 4C and S4 Fig), indicating that defective splicing in the double mutant may be caused by a lack of Cdc28 (PRP2) recruitment or stabilization.

If reduced Cdc28 (PRP2) levels are due to decreased recruitment, we would expect a defect in Cdc28-dependent remodeling of the spliceosome. One way such a defect would manifest itself is by retention of the proteins released or destabilized by Cdc28 (PRP2), including the Nineteen Complex (NTC)-related protein Cwf24 (CWC24) or the RES (REtention and Splicing) factor Cwf26 (BUD13) [37]. We chose to tag and ChIP Cwf24 (CWC24). As expected, in the *Δsnf5sap145* ^*DAm*P^ cells where we saw less Cdc28 (PRP2), we observed much higher levels of Cwc24 in all three genes we looked at, especially at primer sets at or downstream of affected introns (Fig. 4C and S4 Fig). We interpret this to mean that in wild-type cells, Cwf24 is displaced by the Prp2 ATPase and is thus not in the vicinity of chromatin to be crosslinked. In the absence of optimal Cdc28 (PRP2) recruitment, the likelihood of Cwf24 (CWC24) remaining part of the pre-catalytic spliceosome increases, and this is borne out by higher Cwf24 ChIP signal in the *Δsnf5sap145* ^*DAm*P^ double mutants.

We chose not to look at SF3 by ChIP directly for two reasons: first, because *sap145* overexpression may change the local concentration of binding partners and complicate ChIP interpretation and second, because SF3 is known to be destabilized but not completely released from the spliceosome [37] and such nuanced rearrangements are likely not detectable by ChIP.

Notably, we do see a modest increase in Cdc28 (PRP2) ChIP in the *sap145* ^DAmP^ single mutant cells and a decrease in Cdc28 ChIP in the *Δsnf5* single mutant cells, similar to the levels in *Δsnf5sap145* ^*DAm*P^ (S4C Fig), providing some mechanistic insight into how each of these mutations individually affects pre-spliceosome activation. We believe that the overabundance of Sap145 results in a greater requirement for Prp2 remodeling, leading to a higher local concentration of the enzyme, but in the absence of Snf5, Prp2 is not recruited as well. When the two mutations are combined, it creates a situation where the cell needs more Prp2 but has less, which leads to poor splicing outcome.

To provide further evidence that the splicing defect in the *Δsnf5sap145* ^*DAmP*^ double mutants occurs at the step of spliceosome activation, we performed ChIP on a HA-tagged U1 snRNP factor, Usp105 (PRP39) as a control. Compared to wild type, we observed little change in U1 snRNP ChIP along the single intron-containing *rps3* gene (Fig. 4D and S4A Fig). The overall lack of U1 ChIP defect is consistent with our genetic data (showing no synthetic sickness between U1 overexpression and *Δsnf5*) and supports a model in which SWI/SNF contributes downstream of early spliceosome assembly.

**Fig 5:**
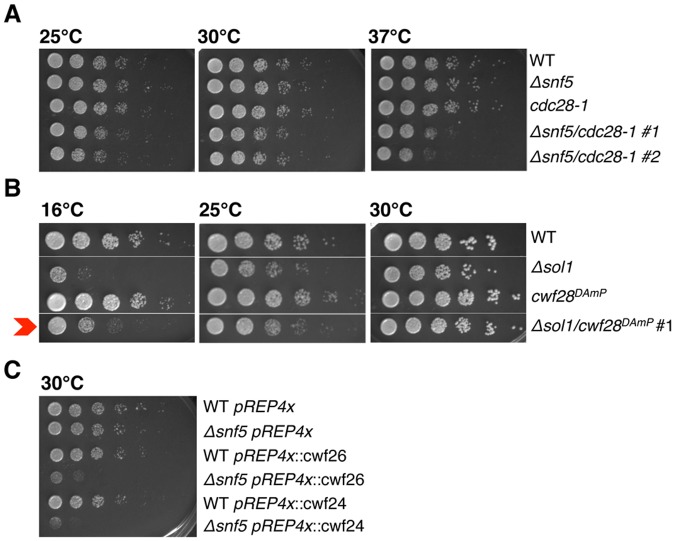
Genetics implicate SWI/SNF at the Prp2 ATPase step of splicing. (A.-C.) Serial dilutions of WT, single and double mutant strains grown at 25°C, 30°C and 37°C (*cdc28-1*), 16°C, 25°C and 30°C (*spp2* ^*DAmP*^) and 30°C (*cwf26* (BUD13) and *cwf24* (CWC24) overexpression). *Δsnf5/cdc28-1 #1*, *#2* represent two independent isolates.

Our ChIP results support a model whereby SWI/SNF contributes to splicing at the Prp2 step of splicing activation. To provide additional lines of evidence to these ends, we again turned to genetics. If our hypothesis that SWI/SNF helps recruit Cdc28 (PRP2) is correct, we would expect that abrogation of Cdc28 (PRP2) function via an alternative method would phenocopy the synthetic sickness seen between SWI/SNF and *sap145* overexpression. We therefore mutated the conserved Gly553 to an Aspartate in *S*. *pombe cdc28* (PRP2), based on the well-characterized *prp2-1* temperature-sensitive allele in *S*. *cerevisiae*, which has been shown to stall splicing after assembly but before first-step catalysis [41,42]. *S*. *pombe cdc28-1* (G553D) was synthetic sick when combined with *Δsnf5*, especially at the non-permissive temperature (37°C) (Fig. 5A), consistent with an increased requirement for SWI/SNF activity at the Prp2 ATPase step.

In *S*. *cerevisiae*, Prp2 is known to act with the cofactor Spp2, an essential G-patch protein that binds to Prp2 and promotes the first step of splicing [43]. Looking back at the *S*. *pombe* splicing E-MAP, we saw that the *cwf28* ^*DAmP*^ (SPP2 in *S*. *cerevisiae*) allele had a strong positive genetic interaction with SWI/SNF (+2.0 and +2.6 with *Δsnf5* and *Δsol1*, respectively; Fig. 1C), suggesting epistasis or suppression. To confirm that *cwf28* and SWI/SNF interacted genetically, we re-created the double mutant by mating, and observed that the *Δsol1/cwf28* ^*DAmP*^ double mutant grew better at 16°C than did the *Δsol1* strain alone, confirming the E-MAP positive genetic interaction (Fig. 5B). In examining the cellular levels of *cwf28* mRNA in the DAmP background, we saw that the DAmP cassette again led to overexpression of *cwf28* (S5 Fig). Interestingly, SPP2 was originally identified as a high-copy suppressor of *prp2-1* [44]. Because SWI/SNF appears to recruit Prp2 and overexpression of Spp2 can overcome the cold-sensitivity of *Δsol1*, we think Spp2 is also acting as a high-copy suppressor of *ΔSWI/SNF*.

To further implicate SWI/SNF in promoting Prp2-dependent spliceosome remodeling, we overexpressed several non-SF3 proteins that are released by Prp2, predicting that their overexpression—like that of SF3—would result in synthetic sickness with *Δsnf5*. Indeed, using the pREP4x vector, ectopic overexpression of *cwf26* (BUD13) and *cwf24* (CWC24) caused a severe growth defect in *Δsnf5* but not wild-type cells (Fig. 5C), consistent with a role for SWI/SNF in promoting Prp2-recruitment and the subsequent release of Cwf26 (BUD13) and Cwf24 (CWC24) from the spliceosome. In summary, we have provided several lines of evidence that SWI/SNF contributes to the Prp2 step of splicing: Prp2 recruitment is defective in *Δsnf5sap145* ^*DAmP*^ cells; SWI/SNF has opposite genetic interactions with *cdc28-1* (*PRP2-1*) and overexpression of *cwf28* (SPP2); and overexpression of *cwf26* and *cwf24* is synthetic sick with *Δsnf5*, presumably due to exacerbating the block in splicing imposed by defective Cdc28 (PRP2) ATPase recruitment.

### Nucleosome positioning is altered in ΔSWI/SNF strains

**Fig 6:**
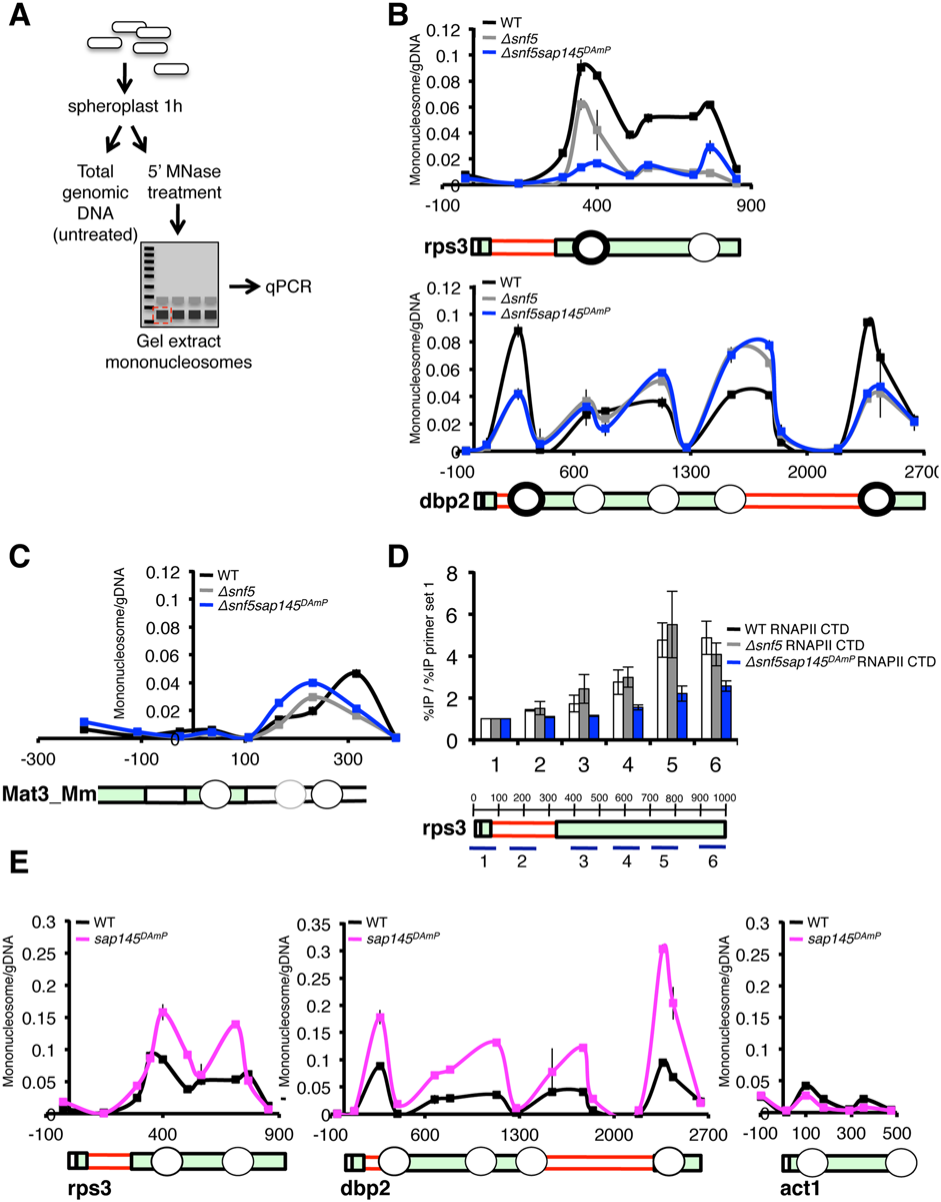
Nucleosome occupancy is altered at intron-containing genes by deletion of snf5 and overexpression of *sap145*. (A.) Schematic representation of nucleosome scanning assay as adapted from [45]. (B.) qPCR analysis of nucleosome occupancy at *rps3* and *dbp2* (intron-containing genes) in WT, *Δsnf5*, *Δsnf5sap145* ^*DAmP*^ strains. Graph is represented as mononucleosome signal/undigested DNA signal at each primer set. Gene representation below is aligned by basepair. Circles represent approximate nucleosome location. Bold circles indicate nucleosomes downstream of introns. (C.) qPCR analysis of nucleosome occupancy at the silenced *mat3_Mm* gene (untranscribed control) in WT, *Δsnf5*, *Δsnf5sap145* ^*DAmP*^ strains. (D.) ChIP performed using anti-RNAPII CTD antibody (8WG16) in WT, *Δsnf5*, and *Δsnf5sap145* ^*DAmP*^ strains. Gene diagram below indicates approximate location of qPCR amplicons and basepairs are shown. Capped error bars are ±SEM of n = 3 biological replicates. (E.) qPCR analysis of nucleosome occupancy at *rps3*, *dbp2* and *act1* (non-intron containing) for WT and *sap145* ^*DAmP*^ strains.

SWI/SNF is generally thought to deposit and remodel nucleosomes on a DNA template in order to regulate RNAPII transcription. To test whether nucleosome positioning and/or occupancy could be altered in *ΔSWI/SNF* cells, we performed a nucleosome-scanning assay, as described in Infante et al., 2012 [45]. Following micrococcal nuclease (MNase) treatment, mononucleosome-protected DNA fragments (~150bp) were excised from an agarose gel and tiling qPCR was performed along several fission yeast genomic loci (Fig. 6A). At all expressed genes examined, we saw a striking decrease in signal from protected DNA in *Δsnf5* and *Δsnf5sap145* ^*DAmP*^ cells, implicating Snf5 in depositing or maintaining the position of nucleosomes along coding regions (Fig. 6B, replicate experiment in S6B, C, D Fig). Both the *Δsnf5* single mutant and the *Δsnf5sap145* ^*DAmP*^ double mutant displayed lower nucleosome occupancy at intron-containing genes, although certain nucleosomes were differentially protected; generally, nucleosomes downstream of introns exhibited the biggest changes in nucleosome occupancy relative to wild type (Fig. 6B, bold circles). We propose that a certain level/distribution of nucleosomes is required for optimal recruitment of Prp2 and that SWI/SNF contributes to creation and/or maintenance of this nucleosome environment at intron-containing genes.

In order to show that the nucleosome occupancy phenotypes we report in *Δsnf5* and *Δsnf5sap145* ^*DAmP*^ cells were not simply due to differences in mononucleosome recovery or MNase accessibility we amplified two regions that we did not expect to be under the control of SWI/SNF: the silenced Mat3-Mm ORF in the mating type locus and an untranscribed gene-poor region. At these two silent regions, we observed no significant change in overall abundance of nucleosomes in the different mutant strains (Fig. 6C and S6B Fig). In our assay, nucleosomes appeared to be less strongly positioned overall (leading to lower signal relative to undigested genomic DNA) at Mat3_Mm and the untranscribed region. This is consistent with published genome-wide data, which show weak nucleosome positioning in these regions during mitotic growth [46].

To correlate the lower nucleosome signal we report at *rps3* and *dbp2* in the *Δsnf5* single mutant and the *Δsnf5/sap145* ^*DAmP*^ double mutant with RNAPII transcription, we examined the ChIP profile of RNA polymerase II, using an antibody against the unmodified RNAPII CTD, antibody (8GW16). RNAPII levels were consistently lower in *Δsnf5/sap145* ^*DAmP*^ strains compared to wild type at almost all points throughout the gene bodies we looked at, even though signal at the promoter was comparable between the two strains (Fig. 6D and S6E Fig). Because this phenotype was strongest in the double mutant, we propose that RNAPII is subject to a level of regulation that relies on both SWI/SNF and splicing being intact. *ΔSnf5* single mutants generally displayed wild-type RNAPII density in ChIP using the 8GW16 antibody (Fig. 6D), although interestingly, additional ChIP experiments using an antibody directed against Ser5 phosphorylated CTD residues (4H8, Abcam) showed reproducibly lower levels of Ser5P CTD in *Δsnf5 and Δsnf5sap145* ^*DAmP*^ strains, consistent with results from Batsche et al., which correlate Ser5P with the SWI/SNF subunit Brm at alternative exons [7]. While it is difficult to garner mechanistic understanding from these results alone, lower RNAPII levels are consistent with lower nucleosome occupancy in the *Δsnf5* and *Δsnf5/sap145* ^*DAmP*^ strains: if nucleosomes act as a barrier to transcription [47,48], then lower nucleosome density should allow for enhanced RNAPII elongation and thus decrease the probability of crosslinking the polymerase molecule to any one point in a gene, though we can not rule out the possibility of changes to RNAPII initiation or processivity.

Surprisingly, we also observed a dramatic increase in nucleosome occupancy in the *sap145* ^*DAmP*^ single mutant cells at intron-containing genes (Fig. 6E). Importantly, when we looked at an intronless gene, *act1*, we did not observe higher nucleosome occupancy in the *sap145* ^*DAmP*^ single mutant, suggesting that *sap145* overexpression is only inducing chromatin changes in the context of splicing at intron-containing genes. These results support a model wherein splicing itself may influence chromatin dynamics. We propose that overexpression of *sap145* inhibits spliceosome activation and that higher levels of nucleosomes serve to compensate for this defect by slowing RNAPII to promote co-transcriptional splicing. Notably, this increase in nucleosome occupancy presumably requires the integrity of the SWI/SNF complex, since, as we describe above, nucleosome occupancy decreased relative to WT when we combined *sap145* overexpression with deletion of *snf5* in our double mutant.

Data from humans has shown that in addition to higher nucleosome abundance in exons vs. introns, nucleosome occupancy further peaks at exons that are flanked by long introns or weak splice sites [49], consistent with our data and with the idea that SWI/SNF-dependent nucleosome positioning is important for pre-mRNA splicing. Taken together, these results point to a role for SWI/SNF in maintaining nucleosome occupancy at expressed genes in *S*. *pombe* and hint at an additional role for splicing factors, or the act of splicing itself, in further promoting nucleosome occupancy.

## Discussion

While a role for chromatin remodeling and histone modifying proteins in regulating alternative splicing has been appreciated for some time, the idea that constitutive splicing decisions also require input from chromatin proteins and RNAPII transcription itself, is just starting to gain traction. Indeed, our Splicing E-MAP is the first high-throughput genetic study to provide evidence that chromatin and transcription have profound connections to pre-mRNA splicing in fission yeast. Here, we provide several lines of evidence to show that the SWI/SNF nucleosome-remodeling complex is important for splicing introns with weak splice sites and contributes to Prp2-dependent spliceosome activation. Importantly, these results demonstrate a role for chromatin in regulating a specific ATP-dependent step of the splicing cycle and provide mechanistic insight into how SWI/SNF may influence RNAPII transcription and splicing through positioning nucleosomes at intron-containing genes.

### A role for chromatin in promoting splicing of introns with weak splices sites

One reason we set out to create a Splicing E-MAP in fission yeast was because of its metazoan-like intron/exon architecture. Over half of intron-containing genes in fission yeast contain more than one intron and the cis-splicing signals in *S*. *pombe* introns are highly variable, in contrast to *S*. *cerevisiae* but mirroring the situation in metazoans. For example, the branch-site consensus sequence in *S*. *cerevisiae* is almost always UACUAAC, whereas the *S*. *pombe* branch-site consensus CURAY (where R is a purine and Y is a pyrimidine), is more similar to that found in mammals (reviewed in [50]). Metazoan splicing decisions are aided by an abundance of SR and hnRNP proteins that regulate exon inclusion or skipping by binding to specific sequences in pre-mRNAs [51]. While the fission yeast genome encodes a small number of SR-like proteins, their roles in intron recognition remain unclear [52,53]. The abundance of degenerate cis-splicing signals combined with the dearth of canonical regulatory proteins in *S*. *pombe* prompts questions of how introns with degenerate splicing signals are recognized in fission yeast and whether there are other pathways to promote co-transcriptional intron recognition and spliceosome assembly. Our results indicate that multiple cellular pathways contribute to efficient splicing in fission yeast, most notably chromatin and RNAPII transcription.

If we take a closer look at the particular chromatin complexes enriched for negative genetic interactions with splicing, we can infer some mechanistic insights. Both SWI/SNF and Ino80 are known to deposit and position nucleosomes in an ATP-dependent manner. Although to date there are no reports implicating Ino80 in splicing, there is solid evidence linking the SWI/SNF catalytic subunit Brm to variant exon inclusion in several human genes. Overexpression of the Brm subunit in human breast cancer cells led to increased exon inclusion in a handful of genes examined and siRNA knockdown of Brm abolished RNAPII accumulation on the same variant exons [7]. The authors of this report proposed SWI/SNF contributes to splicing by decreasing RNAPII elongation rate, thus facilitating co-transcriptional spliceosome recruitment and allowing for recognition of introns with suboptimal spice sites. The idea that a “slow” RNA polymerase promotes spliceosome assembly and recognition of weak splice sites—often referred to as the “kinetic model” of co-transcriptional splicing—is supported by numerous lines of evidence, and seems to be conserved from budding yeast [18] to humans (reviewed in [10]).

Intriguingly, most of the chromatin complexes that were synthetic sick with splicing factors in the E-MAP have been implicated in negative regulation of RNAPII transcription. For example, Set1/Compass, which deposits H3K4 trimethylation has recently been shown, in coordination with H3K4 dimethylation, to repress transcription at coding genes [54]. Similarly, Rpd3L is involved in downregulating transcription at gene promoters by deacetylating histones [55,56]. The cycle of histone acetylation and deacetylation has already been shown to be important for spliceosome recruitment and rearrangements in *S*. *cerevisiae*, wherein deletion of histone deacetylases led to retention of U2 factors and a decrease in recruitment of later splicing factors [16,17]. Taken together, our SWI/SNF data and the Splicing E-MAP as a whole support the general premise of the kinetic model (i.e. repressive chromatin complexes should promote splicing) and provide strong evidence that constitutive splicing decisions also rely on transcriptional inputs.

### Evidence for crosstalk between nucleosome occupancy and splicing across intron-containing genes

**Fig 7:**
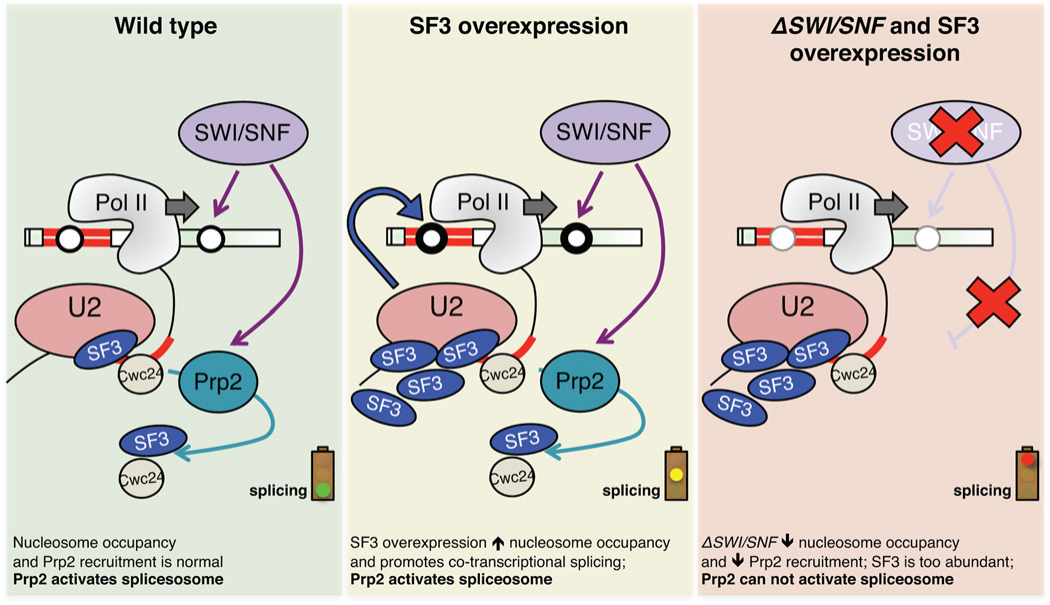
Model illustrating the effect of SF3 overexpression and deletion of SWI/SNF on splicesome activation, nucleosome occupancy and RNAPII elongation. Green panel: Cartoon depicting co-transcriptional recruitment of the Prp2 ATPase to a nascent transcript via SWI/SNF, either directly or via effects of nucleosome deposition on RNAPII dynamics. Optimal recruitment of Prp2 ensures destabilization/release of SF3 and related factors like Cwc24, allowing for activation of the spliceosome. Yellow panel: When SF3 is overexpressed, it creates a greater demand for Prp2 ATPase, stalling the activation of the pre-catalytic spliceosome. Through an unknown feedback mechanism, nucleosome occupancy increases in these conditions in order to “slow down” polymerase and allow time for splicing to occur co-transcriptionally. Red panel: In the absence of SWI/SNF, nucleosome occupancy is decreased along intron-containing genes, alleviating barriers to RNAPII elongation and disfavoring co-transcriptional splicing. These changes to chromatin, coupled with lower local concentrations of Prp2, prevent spliceosome activation in the context of SF3 overexpression.

We propose a model whereby SWI/SNF contributes to the deposition and/or maintenance of nucleosomes in the coding regions of expressed genes in fission yeast (Fig. 7). These nucleosomes may influence splicing in several ways: by influencing polymerase speed and/or promoting pausing, by recruiting splicing factors directly or via adapter proteins, or by directing changes to the CTD modification status. Our data correlate lower nucleosome occupancy with a decrease in RNAPII ChIP and we hypothesize that this reflects changes to RNAPII elongation along coding regions. We propose that these SWI/SNF-dependent changes to nucleosomes and consequently to RNAPII, lead to decreased Prp2 recruitment and defective co-transcriptional spliceosome activation.

Our report that deletion of snf5 leads to a decrease in nucleosome occupancy across spliced genes is consistent with recent findings from Tolstorukov et al., who saw a decrease in nucleosome occupancy at promoters in snf5-deficient mammalian cells [57]. Both these results are in some ways at odds with the general view that SWI/SNF removes nucleosomes from DNA to allow RNAPII to elongate, thus activating gene expression. However, the function and composition of SWI/SNF is remarkably complex and there is good evidence for SWI/SNF also having a role in transcriptional silencing, likely through nucleosome positioning [58]. Our results may necessitate a reworking of how we think about activation of gene expression: to date, SWI/SNF was thought to activate gene expression by removing nucleosomes, but perhaps in some cases it does the opposite—by promoting deposition of nucleosomes that downregulate RNAPII elongation and encourage co-transcriptional splicing, leading to greater levels of steady-state processed mRNAs.

Our observations that sap145 overexpression led to higher level of nucleosomes at intron-containing genes was at first perplexing in light of the fact that we did not see a splicing defect in this strain. However, upon further contemplation, we contend that this nucleosome phenotype is actually compensation for the defect conferred by SF3 overexpression. Overexpression of SF3 alone is deleterious to the cell, in that it disrupts dynamics between SF3b, SF3a and Prp5 interactions [30] and likely interferes with Prp2-dependent spliceosome activation (this work). We argue that this defect is sensed and, through an unknown feedback mechanism, leads to an increase in nucleosome deposition or stabilization of existing nucleosomes in order to inhibit RNAPII elongation and re-establish the timing and balance of co-transcriptional splicing. We believe that this “feedback” is dependent on SWI/SNF and that nucleosome occupancy cannot be restored in the absence of SWI/SNF (accounting for the low nucleosome signal observed when you combine the *sap145* overexpression with deletion of *snf5* (i.e. the double mutant phenocopies the *Δsnf5* single mutant). These data fit nicely with reports from the Beggs and Neugebauer labs, both of which report RNAPII accumulation downstream of introns in *S*. *cerevisiae* [59,60]. The Beggs lab went on to show, quite elegantly, that mutating the branch point sequence in a reporter gene intron led to an accumulation of RNAPII around the 3’SS [60]. They refer to this accumulation as an RNAPII “pause” and propose that splicing itself acts as a checkpoint to stall RNAPII and promote co-transcriptional splicing. We propose that the presence of a nucleosome at or near the 3’SS could contribute to the RNAPII pause reported by the Beggs lab and also believe that splicing itself provides this checkpoint.

One outstanding question is how SWI/SNF and nucleosome occupancy directly impact RNA polymerase II elongation through open reading frames. While our ChIP data are consistent with a faster moving RNAPII decreasing ChIP signal, it is difficult to provide direct evidence of these dynamics *in vivo*. One line of experimentation that could address this question is combining SWI/SNF-SF3 double mutants with RNAPII “fast” and “slow” mutants that have been made and characterized in *S*. *cerevisiae* [18,61]. We would predict that if a slow polymerase mutant could phenocopy a paused polymerase at spliced genes, it would also alleviate the splicing defects seen in the double mutant. However, these mutants have yet to be made and validated in fission yeast.

The multiple connections between the splicing and chromatin machineries, and the specific regulatory connection we show here, support the exciting hypothesis that each of the ATP-dependent steps of the splicing cycle could be regulated by a separate input—which may come from different chromatin remodeling or histone modifying complexes. Implications for such interplay are of great consequence, as mutations in both splicing and chromatin factors—SWI/SNF in particular—are known to promote tumorigenesis in metazoans [62]. Understanding connections between these pathways will be essential not only to elucidate pre-mRNA splicing mechanism, but also to create a full picture of tumor suppression.

## Materials and Methods

### Strains

A list of strains used in and made for this study are available in S2 Table.

### E-MAP analysis

Analysis and generation of the E-MAP data was performed as previously described (Ryan et al., 2012, Roguev et al., 2008[63]). To identify splicing clusters, we performed a thresholded version of UPGMA hierarchical clustering using Pearson’s correlation as the similarity metric. Initially each cluster contains single gene. Clusters were merged as long as the average Pearson correlation between their members was >0.3. Clusters generated using this approach are presented in Fig. 1 and S1 Table.

To evaluate links between splicing factors and processes or complexes (Fig. 1B,D) we used the permutation method of Bandyopadhyay et al [64]. A p-value was calculated by comparing the mean observed interaction score between two groups to that expected by drawing 10^6^ equal-sized random samples of interactions from the E-MAP. All p-values were corrected using the Bonferonni method.

### RNA isolation and RT-qPCR

Cultures were grown overnight in YES5 to saturation and were diluted to 0.005 OD_600_ to allow for overnight growth to mid log-phase. Strains were harvested by centrifugation between 0.5–0.8 OD_600_. Pellets were either snap-frozen in liquid nitrogen or immediately processed for RNA isolation. Total cellular RNA was isolated using hot acid phenol followed by isopropanol precipitation as described in (Bergkessel et al., 2011). Capped error bars represent Standard Error of the Mean (SEM) of 2–4 biological replicates. Uncapped error bars represent 3 technical replicates of a single biological sample.

### Microarrays

*S*. *pombe* splicing-specific microarrays were designed by, and were a generous gift from, Jeffrey Pleiss. The arrays were designed to measure the relative (mutant vs. wild type) amount of every intron and constitutive junction in fission yeast. A single exon probe is used as a measure of total expression for each intron-containing gene. Microarray experiments were performed as outlined in Inada and Pleiss, 2010. Briefly, RNA was isolated as described above. cDNA synthesis was performed according to the manufacturer’s protocol (Invitrogen, SuperScript III) but with slight modifications: 5 mg/ml dN9 random primers were added to 20 ug RNA per synthesis reaction. Reactions were left at 42°C for at least 4 hours. RNA was removed from samples by NaOH treatment and cDNA was labeled with Cy3 (wild-type) and Cy5 (mutant) dyes. Labeled cDNAs were hybridzed to Agilent arrays. Agilent protocols were followed for hybridization and washing conditions.

### Microarray analysis

Splicing changes were expressed as (intron*exon)/junction, to take into account changes to overall gene expression for a given transcript. The R package limma was used to analyze the arrays and the data was normalized by loess normalization. To identify changes to cis-splicing signals that increased the likelihood of an intron being retained in our mutants, intron features were compiled and nucleotides were classified into common (>50% of introns in the fission yeast genome contain that nucleotide at that position) or rare (all other bases). Log odds and their standard errors were calculated based on a logistic regression model for each nucleotide position.

### Chromatin immunoprecipitation (ChIP)

*S*. *pombe* strains were diluted to 0.005 OD_600_ in 50–100 mL of YES5 media and allowed to grow overnight. Cultures were crosslinked in 1% formaldehyde for 15 minutes when they had reached mid-long phase OD_600_ 0.3–0.8. Formaldehyde was quenched with 125 mM glycine for 5 min. Crosslinked cells were spun down at 300 rpm and washed twice with cold dH_2_O. Pellets were flash frozen in liquid nitrogen and stored at -80°C or were immediately processed for ChIP. ChIP was performed as described in Kress et al., 2008, with certain changes for *S*. *pombe*: protease inhibitors were increased (1 mM PMSF, 20 μg/mL leupeptin), bead beating was increased to 1.5’ x 10 cycles and sonication was increased to 6 cycles on high (10 min, 30 sec ON, 1 min OFF). Total and IPed DNA was analyzed by qPCR as described above. Capped error bars represent Standard Error of the Mean (SEM) of 2–4 biological replicates. Uncapped error bars represent 3 technical replicates of a single biological sample.

### Nucleosome scanning assay

Mononucleosomes were prepared as described in Infante et al., 2012, except for the following changes. MNase treatment was performed for 5 minutes using 150U MNase, which resulted in >80% mononucleosome-sized fragments as assayed by agarose gel electrophoresis. Crosslinking was reversed in 1% SDS at 65°C overnight in the presence of Proteinase K (30μl of 30mg/ml). RNase treatment (10μl of 10mg/ml) followed. Total and mononucleosome DNA was analyzed by qPCR as described above using overlapping tiling primer sets.

## Supporting Information

S1 Fig Additional SF3 factors have negative genetic interactions with SWI/SNF. (A.) Pie graphs representing all annotated chromatin complexes in *S*. *pombe*, how many components are in each complex and how many of those components are represented in the non-essential deletion library (green = in library; blue = absent from library) (B.) Heatmap showing genetic interactions from E-MAP between *sap114* (PRP21) and *prp10* (HSH155) with the SWI/SNF complex. Bright blue indicates negative genetic interactions (< -2.5); bright yellow indicates positive (> +2); grey indicates no data.
(TIF)

S2 Fig Splicing defects in SWI/SNF-SF3 double mutants are highly correlated. (A.) Average linkage clustering (using Cluster 3.0) of affected introns as measured by their splicing score ((intron*exon)/junction). Yellow indicates worse splicing (intron retention); blue indicates enhanced splicing. Numbers above heatmap indicate correlation coefficient. (B.) Number of introns whose splicing is defective (yellow) or enhanced (blue) for the single (*Δsnf5*, *Δsol1*, *sap145* ^*DAmP*^, *sap62* ^*DAmP*^) and double mutants (*Δsnf5 sap62* ^*DAmP*^, *Δsol1 sap62* ^*DAmP*^, *Δsnf5 sap145* ^*DAmP*^, *Δsol1 sap145* ^*DAmP*^. Data are expressed as the average log fold change (logFC) log2 ratio of ((intron*exon)/junction) from 2–4 biological replicates. A significance threshold of logFC > 0.5 was chosen.
(TIF)

S3 Fig Overexpression of *sap145* does not cause synthetic sickness in other chromatin mutants. (A.) Growth of WT, *Δpht1*, *Δswr1*, and *Δset1* yeast overexpressing sap145 at 30°C. (B.) RT-qPCR of expression levels conferred by the pREP4x vector of various SF3 factors in WT and *Δsnf5* strain backgrounds. Bars are SF3 factor mRNA signal in pREP4x relative to level in a matched strain background expressing an empty vector, normalized to *act1* mRNA levels. Severity of the growth defect prevented us from harvesting RNA from overexpression strains grown in inducing (minus thiamine) conditions. Therefore, experiment was conducted using yeast grown with thiamine (essentially giving a measure of the “leakiness” of the nmt1 promoter). n = average of 3 technical replicates. (C.) RT-qPCR of uap2(CUS2) expression levels in DAmP strain. Uap2 mRNA levels in WT and uap2^DAmP^ strains were normalized to act1 mRNA levels. (D.) Growth of SWI/SNF-Prp5-DPLD double mutants. *Prp5-DPLD* (*Prp11* in *S*. *pombe*) mutants from the Query lab are deficient in Prp5’s ability to bind SF3b. Each amino acid in the motif was changed to alanine, creating mutants of varying severity (DALD, DPAD). DPLD mutants were combined with *Δsnf5* and *Δsol1* strains and single and double mutants were grown at 30°C.
(TIF)

S4 Fig Cdc28 (PRP2) recruitment and Cwf24 (CWC24) release is impaired in *Δsnf5sap145* ^*DAmP*^ cells. (A.) ChIP using αHA antibodies to detect Cdc28HA (PRP2), Cwf24HA (CWC24) and Usp105HA (PRP39) at SPBC660.16 in WT and *Δsnf5sap145* ^*DAmP*^ cells. Bars represent % IP over % IP at primer set 1 for each amplicon. Red introns are poorly spliced by microarray, RT-qPCR or both. Error bars are ±SEM, n = 3 biological replicates (Usp105), n = 3 technical replicates (Cdc28), n = 2 biological replicates (Cwc24). (B.) ChIP using αHA antibodies to detect Cdc28HA (PRP2), Cwf24HA (CWC24) and Usp105HA (PRP39) at dbp2 in WT and *Δsnf5sap145* ^*DAmP*^ cells. Bars represent % IP over % IP at primer set 1 for each amplicon. Error bars are ±SEM, n = 3 biological replicates (Usp105), n = 2 biological replicates (Cdc28), n = 3 technical replicates (Cwc24). (C.) ChIP using αHA antibodies to detect Cdc28HA (PRP2) along rps3 in WT, *sap145* ^*DAmP*^, *Δsnf5* and *Δsnf5sap145* ^*DAmP*^ cells. Error bars are ±SEM, n = 3 technical replicates.
(TIF)

S5 Fig Cwf28 (SPP2) DAmP allele is overexpressed. RT-qPCR of cwf28 (SPP2) mRNA in WT, single and double mutants. Bars represent cwf28 signal normalized to Atf1 mRNA. Error bars are ±SEM, n = 3 technical replicates.
(TIF)

S6 Fig Nucleosome occupancy is altered by deletion of SWI/SNF and overexpression of sap145. (A.-D.) Biological replicates of experiment shown in Fig. 6. Data are represented as mononucleosome signal/undigested DNA signal at each primer set. Gene representation below is aligned by basepair. Circles represent approximate nucleosome location. Bold circles indicate nucleosomes downstream of introns. Colored background rectangles are used to group panels looking at the same gene locus (light purple = *rps3*; pink = gene poor region; beige = *dbp2*; light blue = *act1*). (E.) ChIP using antibodies against RNAPII CTD (antibody [8WG16], Neoclone) in WT, *Δsnf5* and *Δsnf5sap145* ^*DAmP*^ cells at the *dbp2* gene. Error bars are ±SEM, n = 3 biological replicates (F.) ChIP using antibodies against RNAPII Ser5P CTD (antibody [4H8], Abcam) in WT, *Δsnf5* and *Δsnf5sap145* ^*DAmP*^ cells at *dbp2* and *rps3* genes. Data are shown as the change relative to WT (which is shown as 1). Error bars are ±SEM, n = 3 biological replicates. (G.) Biological replicates of experiment shown in Fig. 6E at rps3, dbp2 and act1 genes. Basepair alignments are indicated at the top of each graph.
(TIF)

S1 Table Pairwise genetic interaction scores for splicing E-MAP. Splicing factor strains are on y-axis; library deletion strains are on x-axis.
(ZIP)

S2 Table List of all splicing strains generated for splicing E-MAP, organized by snRNP. Clusters, as introduced in Fig. 1a are also detailed. *S*. *cerevisiae* orthologs are provided.
(ZIP)

S3 Table Strain list. Detailed list of all strains described in this paper.
(ZIP)
