# Supplementary figures and images for: Genetic Interaction Mapping Reveals a Role for the SWI/SNF Nucleosome Remodeler in Spliceosome Activation in Fission Yeast
Source: PLoS Genet. 2015 Mar 31;11(3):e1005074. doi: 10.1371/journal.pgen.1005074 (PMC4380400; doi:10.1371/journal.pgen.1005074)

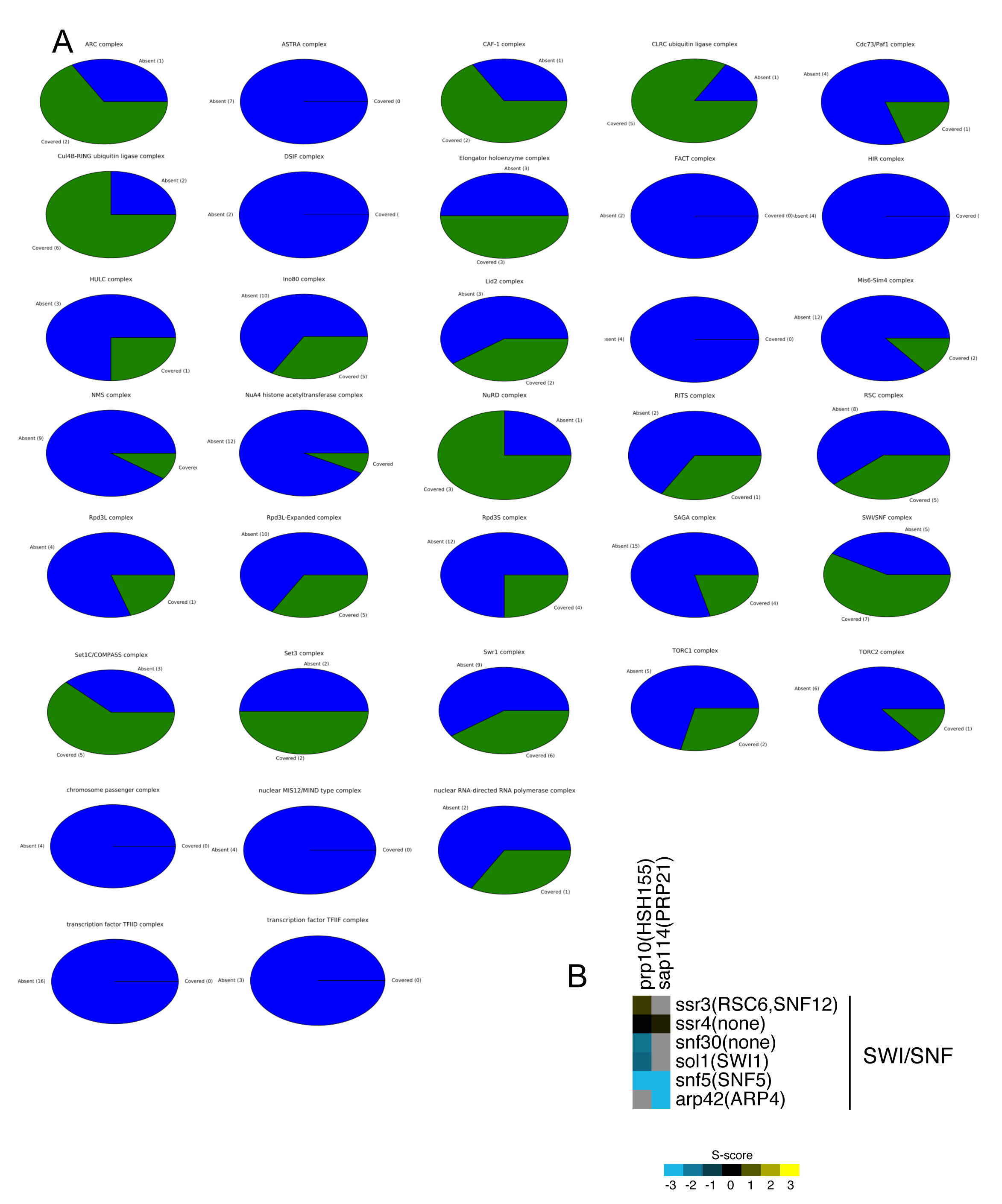

Supplement: S1 Fig — (A.) Pie graphs representing all annotated chromatin complexes in S. pombe, how many components are in each complex and how many of those components are represented in the non-essential deletion library (green = in library; blue = absent from library) (B.) Heatmap showing genetic interactions from E-MAP between sap114 (PRP21) and prp10 (HSH155) with the SWI/SNF complex. Bright blue indicates negative genetic interactions (< -2.5); bright yellow indicates positive (> +2); grey indicates no data. (TIF) [file pgen.1005074.s001.tif]

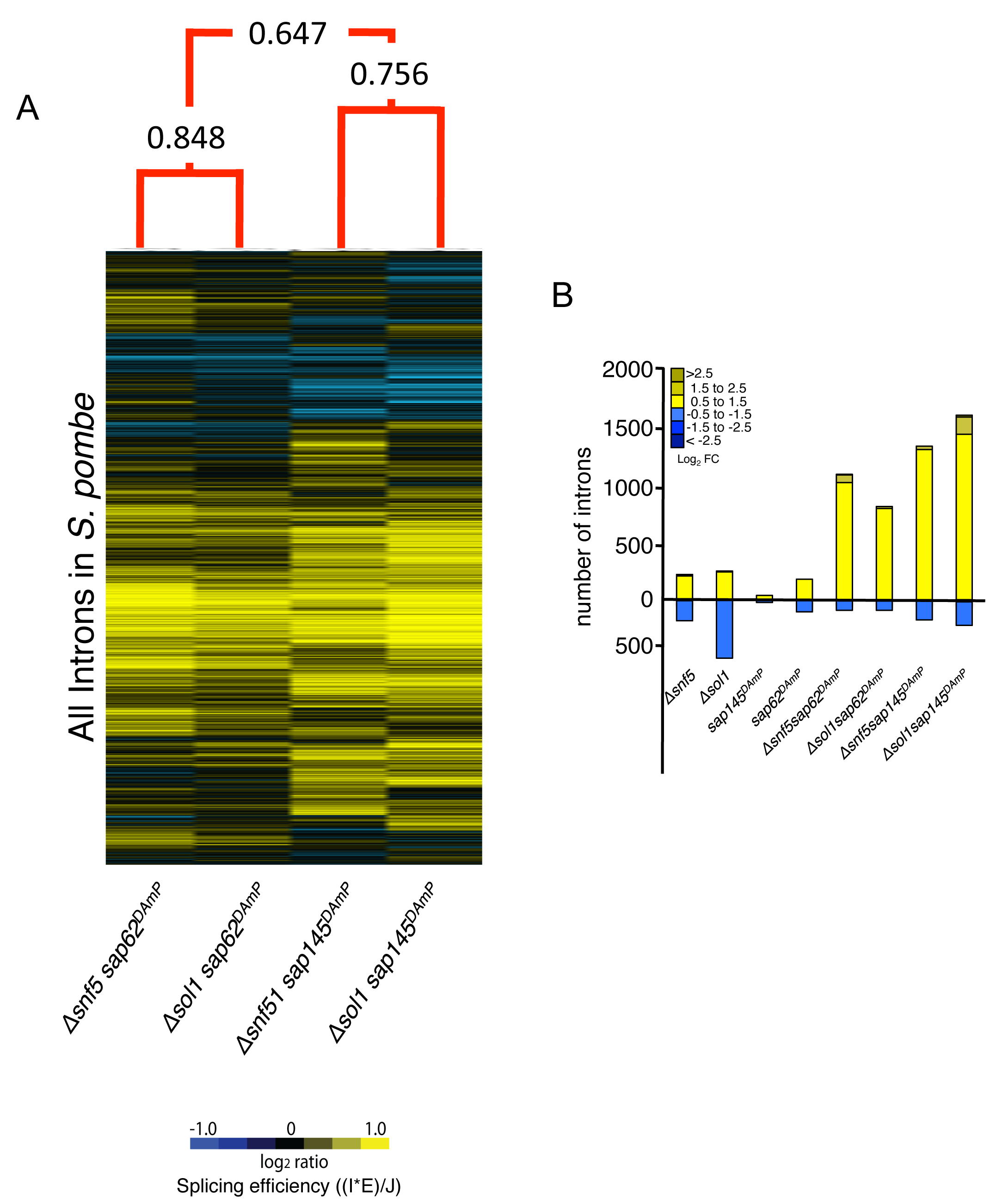

Supplement: S2 Fig — (A.) Average linkage clustering (using Cluster 3.0) of affected introns as measured by their splicing score ((intron*exon)/junction). Yellow indicates worse splicing (intron retention); blue indicates enhanced splicing. Numbers above heatmap indicate correlation coefficient. (B.) Number of introns whose splicing is defective (yellow) or enhanced (blue) for the single (Δsnf5, Δsol1, sap145 DAmP, sap62 DAmP) and double mutants (Δsnf5 sap62 DAmP, Δsol1 sap62 DAmP, Δsnf5 sap145 DAmP, Δsol1 sap145 DAmP. Data are expressed as the average log fold change (logFC) log2 ratio of ((intron*exon)/junction) from 2–4 biological replicates. A significance threshold of logFC > 0.5 was chosen. (TIF) [file pgen.1005074.s002.tif]

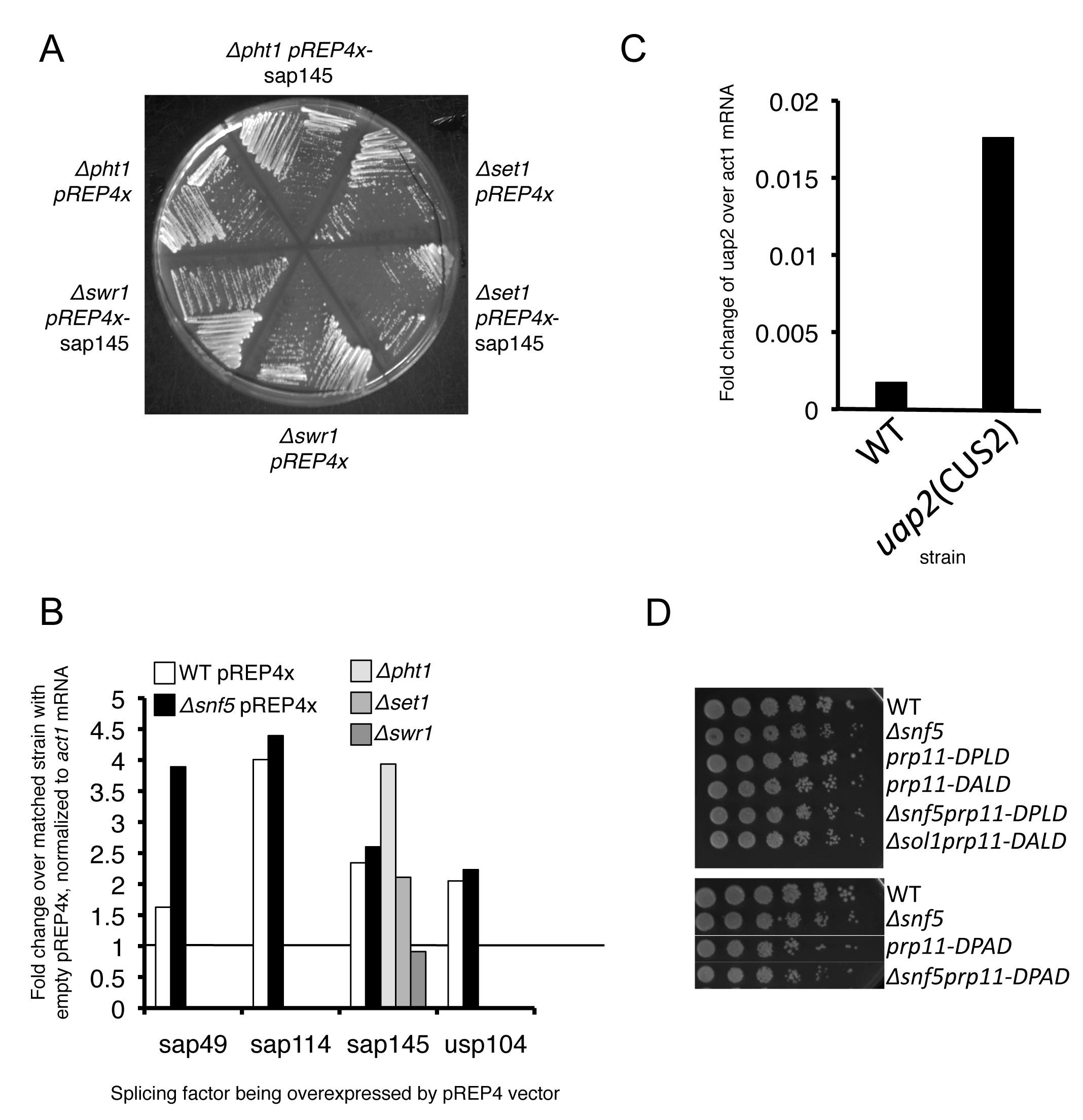

Supplement: S3 Fig — (A.) Growth of WT, Δpht1, Δswr1, and Δset1 yeast overexpressing sap145 at 30°C. (B.) RT-qPCR of expression levels conferred by the pREP4x vector of various SF3 factors in WT and Δsnf5 strain backgrounds. Bars are SF3 factor mRNA signal in pREP4x relative to level in a matched strain background expressing an empty vector, normalized to act1 mRNA levels. Severity of the growth defect prevented us from harvesting RNA from overexpression strains grown in inducing (minus thiamine) conditions. Therefore, experiment was conducted using yeast grown with thiamine (essentially giving a measure of the “leakiness” of the nmt1 promoter). n = average of 3 technical replicates. (C.) RT-qPCR of uap2(CUS2) expression levels in DAmP strain. Uap2 mRNA levels in WT and uap2DAmP strains were normalized to act1 mRNA levels. (D.) Growth of SWI/SNF-Prp5-DPLD double mutants. Prp5-DPLD (Prp11 in S. pombe) mutants from the Query lab are deficient in Prp5’s ability to bind SF3b. Each amino acid in the motif was changed to alanine, creating mutants of varying severity (DALD, DPAD). DPLD mutants were combined with Δsnf5 and Δsol1 strains and single and double mutants were grown at 30°C. (TIF) [file pgen.1005074.s003.tif]

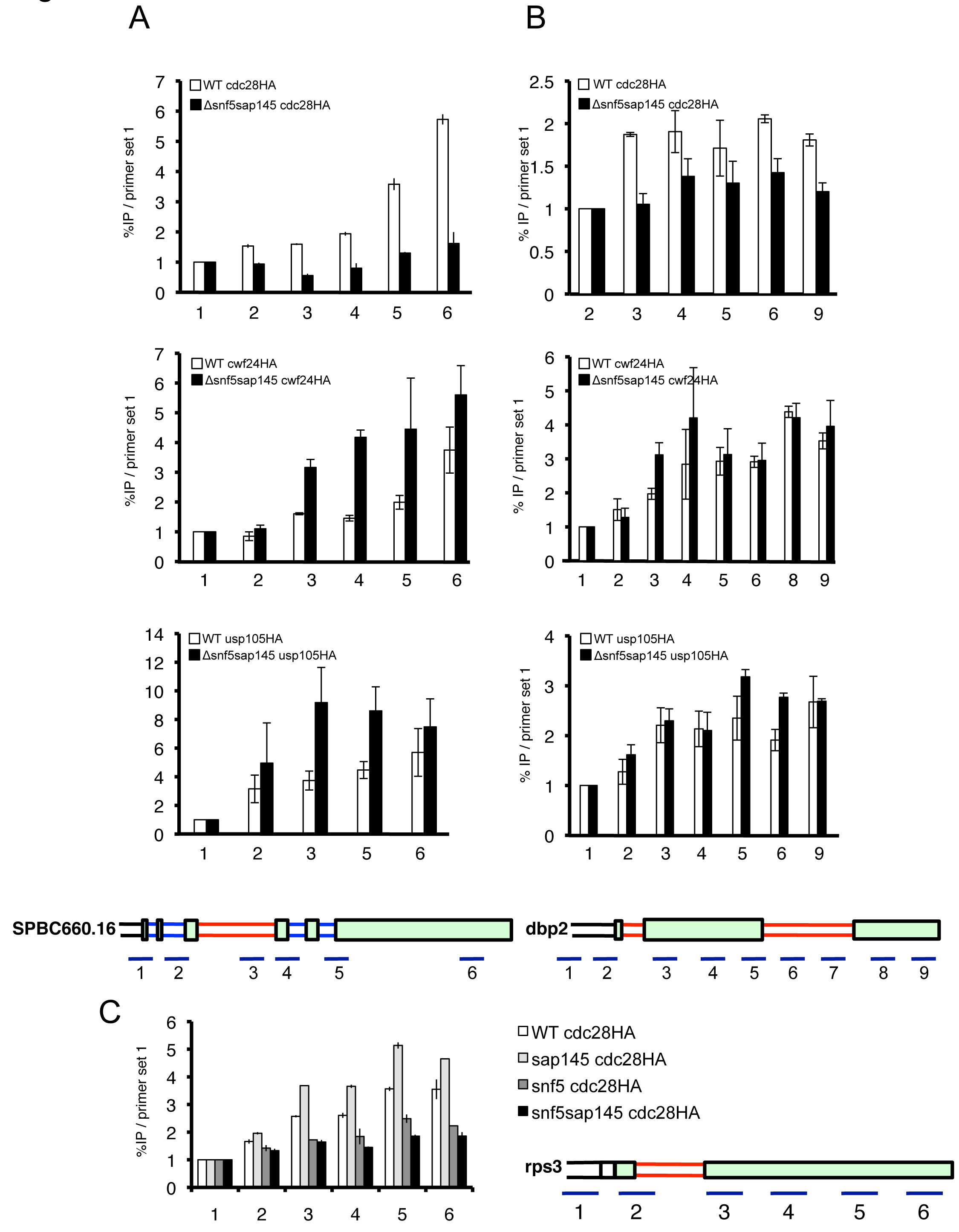

Supplement: S4 Fig — (A.) ChIP using αHA antibodies to detect Cdc28HA (PRP2), Cwf24HA (CWC24) and Usp105HA (PRP39) at SPBC660.16 in WT and Δsnf5sap145 DAmP cells. Bars represent % IP over % IP at primer set 1 for each amplicon. Red introns are poorly spliced by microarray, RT-qPCR or both. Error bars are ±SEM, n = 3 biological replicates (Usp105), n = 3 technical replicates (Cdc28), n = 2 biological replicates (Cwc24). (B.) ChIP using αHA antibodies to detect Cdc28HA (PRP2), Cwf24HA (CWC24) and Usp105HA (PRP39) at dbp2 in WT and Δsnf5sap145 DAmP cells. Bars represent % IP over % IP at primer set 1 for each amplicon. Error bars are ±SEM, n = 3 biological replicates (Usp105), n = 2 biological replicates (Cdc28), n = 3 technical replicates (Cwc24). (C.) ChIP using αHA antibodies to detect Cdc28HA (PRP2) along rps3 in WT, sap145 DAmP, Δsnf5 and Δsnf5sap145 DAmP cells. Error bars are ±SEM, n = 3 technical replicates. (TIF) [file pgen.1005074.s004.tif]

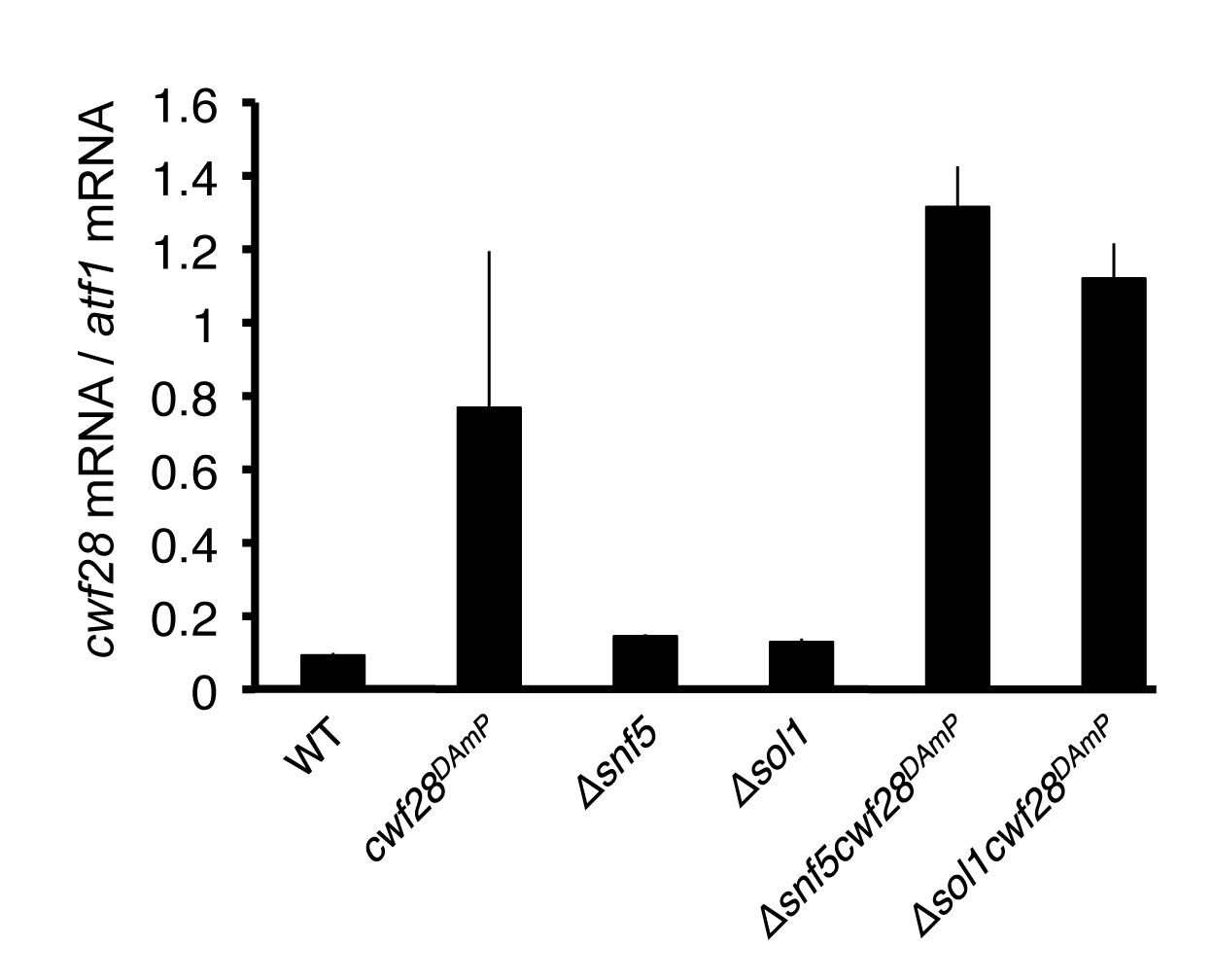

Supplement: S5 Fig — RT-qPCR of cwf28 (SPP2) mRNA in WT, single and double mutants. Bars represent cwf28 signal normalized to Atf1 mRNA. Error bars are ±SEM, n = 3 technical replicates. (TIF) [file pgen.1005074.s005.tif]

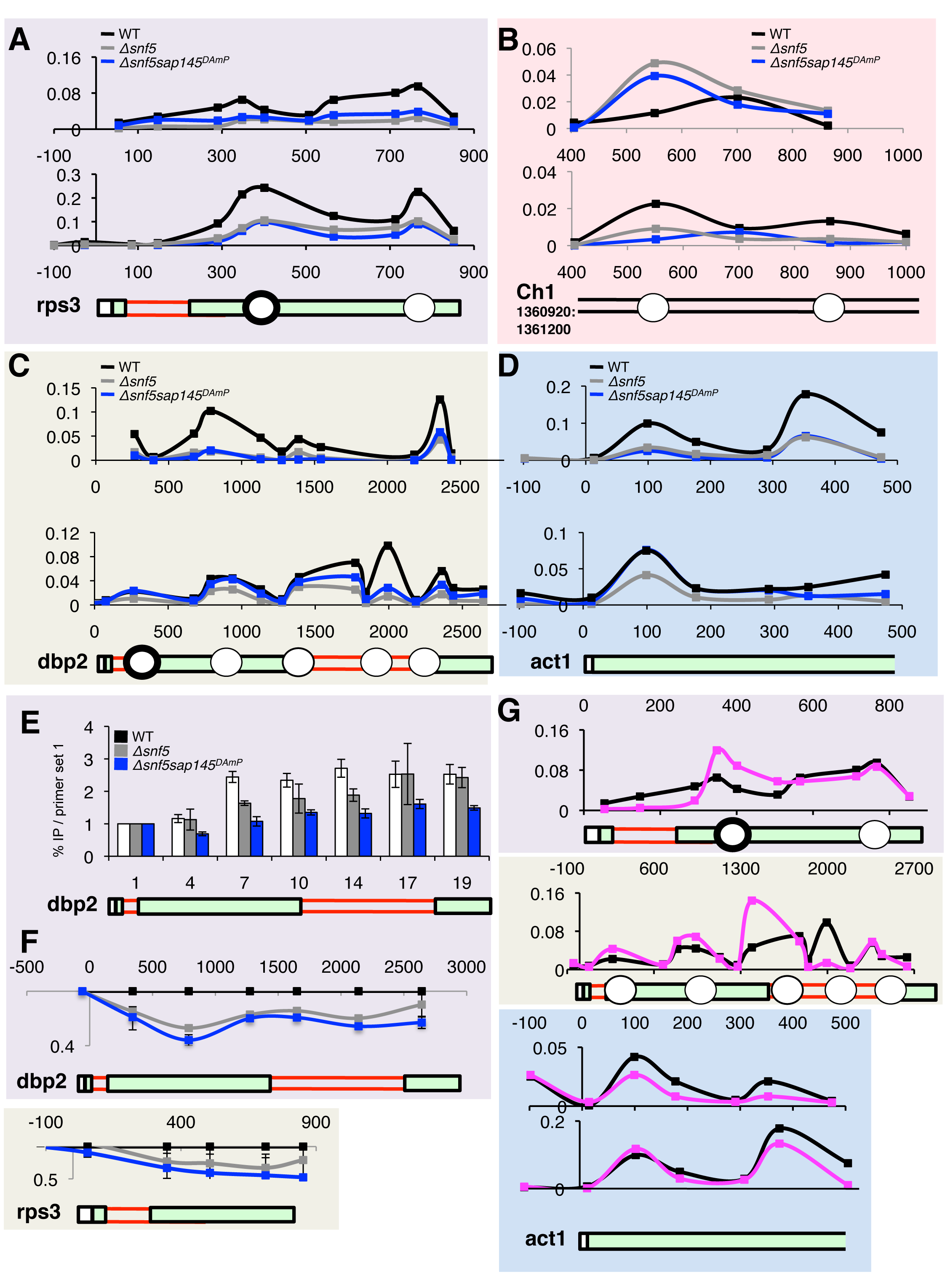

Supplement: S6 Fig — (A.-D.) Biological replicates of experiment shown in Fig. 6. Data are represented as mononucleosome signal/undigested DNA signal at each primer set. Gene representation below is aligned by basepair. Circles represent approximate nucleosome location. Bold circles indicate nucleosomes downstream of introns. Colored background rectangles are used to group panels looking at the same gene locus (light purple = rps3; pink = gene poor region; beige = dbp2; light blue = act1). (E.) ChIP using antibodies against RNAPII CTD (antibody [8WG16], Neoclone) in WT, Δsnf5 and Δsnf5sap145 DAmP cells at the dbp2 gene. Error bars are ±SEM, n = 3 biological replicates (F.) ChIP using antibodies against RNAPII Ser5P CTD (antibody [4H8], Abcam) in WT, Δsnf5 and Δsnf5sap145 DAmP cells at dbp2 and rps3 genes. Data are shown as the change relative to WT (which is shown as 1). Error bars are ±SEM, n = 3 biological replicates. (G.) Biological replicates of experiment shown in Fig. 6E at rps3, dbp2 and act1 genes. Basepair alignments are indicated at the top of each graph. (TIF) [file pgen.1005074.s006.tif]
